# *In Silico* Structural Modeling of the HuR-mRNA Complex: Insights into Structural and Functional Regulation

**DOI:** 10.1021/acs.jcim.5c01028

**Published:** 2025-10-01

**Authors:** Davide Pietrafesa, Alice Romeo, Fabio Giovanni Tucci, Paola Fiorani, Federico Iacovelli, Mattia Falconi

**Affiliations:** † Department of Biology, 9318 University of Rome “Tor Vergata”, Via della Ricerca Scientifica 1, 00133 Rome, Italy; ‡ Institute of Translational Pharmacology, National Research Council, CNR, Via del Fosso del Cavaliere 100, 00133 Rome, Italy

## Abstract

The RNA-binding protein HuR (embryonic lethal abnormal vision-like protein 1) regulates mRNA stability and translation. HuR contains three RNA-recognition motifs (RRMs): the RRM1 and RRM2 confer high-affinity mRNA binding, while RRM3 mediates protein oligomerization. Although HuR is predominantly nuclear, cellular stimuli trigger its cytoplasmic translocation *via* a nucleocytoplasmic shuttling sequence between the RRM2 and RRM3 domains. Despite HuR’s critical role in post-transcriptional gene regulation, its full-length three-dimensional (3D) structure remains uncharacterized. In this study, we employed an *in silico* approach, combining molecular modeling, atomistic, and coarse-grained molecular dynamics simulations to build and validate a 3D model of the full-length HuR in complex with an mRNA fragment. Structural analysis of the model identified a tyrosine residue as a key mediator of HuR-RNA interaction stability and provided novel structural insights into HuR’s RNA-binding mechanism, contributing to a deeper understanding of its regulatory functions.

## Graphical abstract

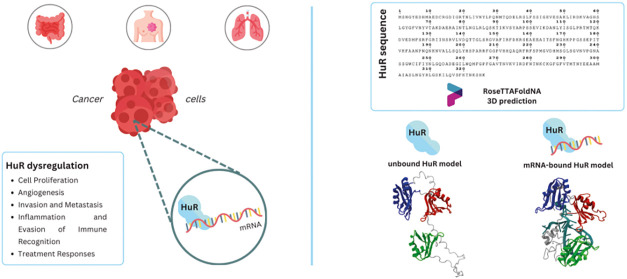

## 1 Introduction

Gene regulation is essential for all RNA classes’ maturation, transport, stability, and degradation, enabling cells to respond to internal and external stimuli. RNA-binding proteins (RBPs) are trans factors that play an essential role in gene expression regulation, binding to specific *cis* elements of target mRNAs (mRNAs).^1^ Within this regulatory network, RBPs modulate nuclear RNA processes, including splicing, capping, polyadenylation, and the cytoplasmic fate of mRNA.^2^

Among the most studied RBPs, Human Antigen R (HuR, also named ELAV-like protein 1) is a ubiquitously expressed RBP from the embryonic lethal abnormal vision (ELAV)-like protein family. HuR binds to adenine- and uridine-rich elements (ARE), principally located in the mRNA 3′-untranslated region (UTR),^3^ which encodes oncoproteins, cytokines, growth factors, and transcription factors.^4–8^

HuR is a multidomain protein of 326 amino acids with a molecular weight of approximately 36 kDa. It contains three RNA-recognition motifs (RRM): the N-terminal domains RRM1 (residues 20–98) and RRM2 (residues 106–186), which recognized U-rich hairpin loops, and the C-terminal domain RRM3 (residues 244–322), that facilitates interaction with the mRNA, binding to a polyA tail.^9^ RRM1 and RRM2, linked by a short, 8-residue sequence, are the primary determinants of HuR’s high-affinity mRNA binding. RRM3, with an essential hinge region connecting it to RRM2, drives the cooperative assembly of HuR oligomers on RNA.^9^

The RRMs adopt an αβ-sandwich topology consisting of a β1α1β2β3β2β4 structure with a β-sheet formed by four antiparallel β-strands folded against two α-helices. Although structurally similar, RRM1 and RRM2 exhibit a distinct conformation due to a β-hairpin located in the loop between α2 and β4. In RRM1, this region adopts a β-turn-β conformation, which is absent in RRM2. The two central β-strands (β1 and β3) in each RRM contain two highly conserved ribonucleoprotein motifs, RNP1 and RNP2, crucial for binding to the target mRNA.^10^

HuR’s function is regulated at multiple levels. First, its cellular concentration is modulated through transcription, polyadenylation, and mRNA stability mechanisms.^11,12^ Furthermore, post-translational modifications, such as phosphorylation, ubiquitination, neddylation, and caspase-mediated cleavage, have been demonstrated to influence HuR’s cellular levels and localization.^13^ Additionally, HuR’s binding to target mRNAs is regulated by phosphorylation, methylation, and ubiquitination, affecting its RNA-binding, subcellular localization, and stability.^13–15^

Although HuR is primarily a nuclear protein, it rapidly translocates to the cytoplasm in response to specific stimuli, mediated by a nucleocytoplasmic shuttling sequence (HSN) located in the RRM2–RRM3 hinge region.^16^ HuR can coordinate the turnover of target mRNAs involved in stress response in the cytoplasm, ensuring cell survival.^16^

To carry out their function, all the ELAV proteins have been shown to form dimers and multimers on RNA targets, with the involvement of RRM3 and the essential hinge region.^9,16,17^ Specifically, disruptions at the dimerization interface reduced HuR’s binding affinity for its targets.^18^ The RRM2–RRM3 hinge region may also contribute to dimerization, but the specific role is still unclear.^18^

Full-length HuR exists in a dynamic equilibrium between monomeric and oligomeric states, with the latter becoming more prominent at higher protein concentrations.^18^ The residue W261 in HuR’s RRM3 plays a critical role in mediating this dimerization, and its mutation significantly disrupts both dimerization and oligomerization *in vitro*, affecting the full-length HuR and RRM3.^19^ The dimerization interface is formed by stacking interactions involving W261 and hydrogen bonds (HBs) between residues from helix α1 and the α1−β2 loop.^18^

While HuR plays a protective and antiapoptotic role under normal stress conditions, this function becomes dysregulated in cancer, promoting malignant cell growth, survival, and metastasis.^20^ Indeed, HuR expression levels are significantly elevated in a wide variety of cancer tissues compared to their regular counterparts.^21^ Increased cytoplasmic accumulation of HuR is associated with more aggressive malignancies and serves as a prognostic marker for poor clinical outcomes in cancers of the colon,^21–23^ prostate,^24,25^ breast,^26^ brain,^27^ ovaries,^28^ pancreas,^29^ liver,^10^ and lungs.^30^

The complete three-dimensional (3D) structure of HuR has not yet been resolved. Specifically, only ten partial structures of HuR can be retrieved from the RCSB Protein Data Bank (PDB),^31^ nine of which were resolved by X-ray diffraction and one by NMR spectroscopy. Among these, three include the RRM1 (PDB IDs: 4FXV, 3HI9, 5SZW),^32,33^ two the RRM1–RRM2 domains (PDB IDs: 4ED5, 4EGL)^34^ and five the RRM3 domain (PDB IDs: 6GD2, 6GD3, 6G2K, 6GD1, and 6GC5).^18,35^

To address the lack of a complete structure, we employed a combined computational approach, including modeling, classical and coarse-grained (CG)^36^ molecular dynamics (MD) simulations to generate and assess a 3D model of the HuR-mRNA complex. Analysis of the model provided novel structural insights into the mechanism by which HuR binds RNA and identified a critical role for residue Y26 in stabilizing the HuR-RNA complex, furthering the understanding of its regulatory functions.

## 2 Materials and Methods

### 2.1 Molecular Modeling of HuR-mRNA and HuR complexes

The human HuR protein sequence was obtained from the UniProt database (UniProt ID: Q15717) (Figure 1A), while the RNA sequence 5′-AUAUUUAUAUUUUUAUUUUAUUUUUUU-3′ was retrieved from the work of Ma et al.^7^ Due to the unstable nature of RNA, there are no experimentally solved structures of HuR in complex with RNA in the PDB. Consequently, the 27-nucleotide RNA fragment, corresponding to the sequence of the IL-3 ARE, with binding regions recognized by HuR,^7^ has been used for the modeling procedure. This fragment corresponds to the sequence of the IL-3 ARE, with binding regions consisting of multiple tandem AUUUA motifs characteristic of class II of AU-rich elements, as all the HuR target mRNAs.^6^ Given the aim to model a representative HuR-mRNA complex, which contains intrinsically disordered regions, the IL-3 ARE offered a reliable framework to predict a stable model and capture key protein–RNA molecular interactions, including RRM-uridine contacts and electrostatic complementarity, that are canonical in the HuR-mRNA interaction. The input sequences were provided to RoseTTAFoldNA^37^ for the prediction of HuR and HuR-mRNA complex structures. RoseTTAFoldNA was selected for its demonstrated higher accuracy in modeling protein–RNA complexes that, in the case of HuR-mRNA need to be modeled in a coordinated manner. This decision was based on its excellent performance, particularly with moderate-length nucleic acid sequences and protein sizes.^37^ An iterative modeling procedure was employed, generating 1000 models per structure. This approach balances computational feasibility with the need for different structural explorations, increasing the likelihood of identifying representative and energetically favorable conformations. Model refinement continued until convergence, with a maximum acceptable threshold of 30 atomic clashes per model. The convergence of the models was statistically assessed using a clustering analysis based on structural similarity, employing the GROMOS method of the gmx cluster of GROMACS 2024 suite,^38^ with a cutoff distance of 0.31 nm. This process resulted in the identification of six clusters for the HuR-mRNA system and four clusters for the HuR system. The centroids of the first and most populated cluster for both systems (namely, model numbers 676 and 838) were then extracted and used as an initial structure for starting MD simulations. The quality of the centroids has also been assessed using the ERRAT,^39^ VERIFY,^40,41^ WHATCHECK,^42^ and PROCHECK^43^ validation programs of the UCLA SAVES v6.1 (https://saves.mbi.ucla.edu/). The QMEANDisCo estimation method^44^ of SWISS-MODEL^45^ was also used (https://swissmodel.expasy.org/qmean).

**Figure 1:**
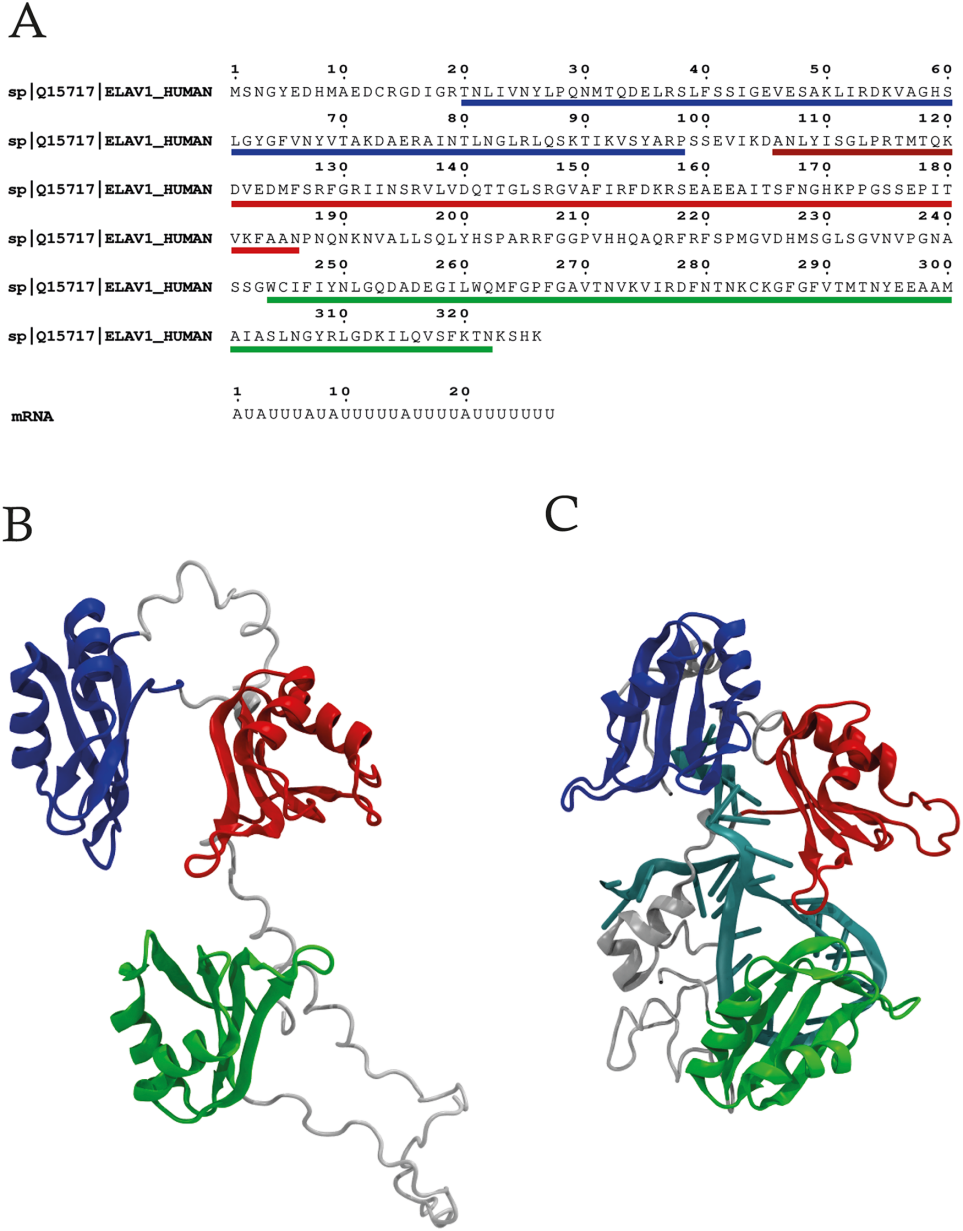
RoseTTAFoldNA modeling. (A) Protein and mRNA fragment sequences. (B) 3D model number 676 of the unbound HuR. (B) 3D model number 838 of HuR-mRNA complex. For both models, the RRM1 (blue), RRM2 (red), and RRM3 (green) are highlighted. The mRNA fragment is colored cyan, while the rest of the protein is in gray.

### 2.2 Classical Molecular Dynamics Simulations

Topologies of the wild-type (WT) and Y26A HuR and HuR-mRNA complexes were generated using the tleap module of the AmberTools23 program.^46^ The AMBER ff19SB^47^ and OL3 ^48^ were used to parametrize the HuR and RNA, respectively. Each complex was inserted in a box of TIP3P water molecules^49^ and neutralized with 32 Na^+^ ions. To address RNA’s spurious self-interaction tendencies arising from force field bias, the stafix OL3 force field modification was applied to the 27 nt of mRNA.^50^

Five minimization cycles were performed to remove unfavorable interactions; each composed of 500 steps of steepest descent followed by 500 steps of conjugate gradient algorithms. A starting restraint of 20 kcal·mol^–1^·Å^–2^ was imposed on the protein and RNA atoms; it was then slowly reduced and removed in the last minimization cycle. The system’s temperature gradually increased from 0 to 310 K in an NVT ensemble using the Langevin thermostat^51^ over a period of 2.0 ns. A starting restraint of 0.5 G·Å^–2^ was imposed on the protein and RNA atoms and then gradually decreased to relax the system slowly. Systems were then simulated in an isobaric–isothermal (NPT) ensemble for 2.0 ns using the Berendsen barostat,^52^ imposing a pressure of 1.0 atm and maintaining the temperature to 310 K. The SHAKE algorithm^53^ was used to constrain bonds involving hydrogen atoms.

The production runs were generated using the module pmemd.cuda module of AMBER23 software,^54^ and the system coordinates were written every 1000 steps. The HuR and HuR-RNA systems were simulated in five independent replicas, each of 500.0 ns.

Long-range interactions were calculated using the PME method,^55^ while a cutoff of 8.0 Å was imposed for short-range interactions. Simulations were executed on a GeForce RTX 4080 GPUs.

### 2.3 Coarse-Grained Molecular Dynamics Simulations

The centroid of the most populated cluster, extracted from the analysis of the HuR-mRNA MD trajectory, was linearized using Chimera^56^ and Pymol software,^57^ removing the mRNA fragment. The linearized model, namely, CG-HuR, was energetically minimized in five steps to remove unfavorable interactions, each composed of 500 steps of steepest descent followed by 500 steps of conjugate gradient algorithms. A starting restraint of 20 kcal·mol^–1^·Å^–2^ was imposed on the protein atoms, then slowly reduced and removed in the last minimization cycle.

The complex was then converted from an atomistic to a CG model using the Martinize2 program,^58^ combined with an elastic model to preserve the tertiary and quaternary structure of the RRMs domains. Complex topology was generated using the Martini 3 CG force field^36^ and the Gromacs 2024 suite.^38^ The complex was inserted in a box of CG water and equilibrated at a temperature of 310 K and a pressure of 1 bar. Seven Na^+^ ions were used to neutralize the system. Three minimization cycles were performed, starting with a restraint of 1000 kcal·mol^–1^·Å^–2^ on the backbone beads, which was reduced to 500 kcal·mol^–1^·Å^–2^ in the second cycle and completely removed in the last one. The CG system was then equilibrated in five cycles of the isothermal–isobaric (NPT) ensemble for 50.0 ns using the Berendsen thermostat,^52^ imposing a pressure of 1.0 atm and maintaining the temperature at 310.0 K. The LINCS algorithm^59^ was used to constrain bonds involving hydrogen atoms. The production runs were generated using the module mdrun of GROMACS 2024 software,^38^ and the system coordinates were written every 5000 steps. The CG-HuR systems were simulated in five independent replicas, each with 10.0 μs. Long-range interactions were calculated using the PME method,^55^ while a cutoff of 8.0 Å was imposed for short-range interactions. Simulations were executed on GeForce RTX 4080 GPUs.

### 2.4 Trajectories Analysis

The trajectories were analyzed with the analysis modules of the GROMACS 2024 suite.^38^ The first 100 ns of all simulations were not considered for the analyses, representing a thermalization phase for the systems (see Supporting Information Figure S2). The root-mean-square deviations (RMSD) and root-mean-square fluctuations (RMSF) were computed using the rms and rmsf modules, respectively. The gyration radius (GR) was calculated using the gyrate module. The principal component analysis (PCA) was performed for each trajectory on the 326 Cα atoms of HuR using the covar and anaeig modules. The dynamical cross-correlation matrices (DCCMs) were retrieved by using an in-house Python script. The relative arrangements of the RRM domains were analyzed by calculating interdomain distances and orientations for each pair (RRM1–RRM2, RRM2–RRM3, and RRM1–RRM3) across simulation trajectories. For each frame, we computed the distance between the centers of mass (COMs) of the respective domains using the gmx mindist tool from GROMACS 2024. Additionally, the interdomains angle was calculated using the angle module of GROMACS by considering the angle between the COMs of the RRM1, RRM2 and RRM3 domains.

The Hbonds plugin of VMD 1.9.3 ^60^ was used to analyze the intraprotein and inter (protein–mRNA) hydrogen bonds network. A hydrogen bond was assumed to exist if the donor–acceptor distance was shorter than 0.30 nm and the hydrogen-donor–acceptor angle was less than 30°.

The π–π base pair stacking was analyzed using an in-house Python script using a distance between aromatic angles (*d*) of 5.5 nm and the angle (α) of stacking of 40°. The C, C5, N1, and N3 atoms of RNA and CG, CE1, CE2, and CZ atoms of HuR were used for this analysis. The minimum distance analysis was conducted using the mindist module of the GROMACS 2024 suite.^38^

Molecular mechanics/Poisson–Boltzmann Born and surface area continuum solvation (MM/PBSA) nonlinear analyses^46^ were performed over each trajectory using the MMPBSA.py.MPI program, implemented in the AMBER23 software,^54^ setting the ionic strength to 0.15 M. Images were rendered using VMD 1.9.3 ^60^ or R 4.4.3.^61^

### 2.5 Analysis on Y26 Conservation

The ConSurf web server (https://consurf.tau.ac.il/consurf_index.php)^62^ was used to evaluate the evolutionary conservation, employing the phylogenetic relations between homologous sequences. The HuR structure, obtained from the HuR-mRNA complex after removal of the mRNA fragment, was used for the calculation. The homologues were collected from the UNIREF90 database,^63^ employing the HMMER method for the homologues search algorithm.^64^ The multiple sequence alignment (MSA) was built using MUSCLE implemented in the MEGA4 program,^65^ and the conservation scores were calculated with the Bayesian method. To assess the presence of a tyrosine residue in position 26, an alignment of the protein sequences of the deposited structures available in the PDB of the ELAV/Hu protein family was performed. The HuC (PDB ID: 1D8Z, 1D9A, and 1FNX)^66^ of *Mus musculus* and HuD (PDB ID: 1FXL, and 1G2E)^67^ of *Homo sapiens* were used. No structures of HuB were found. The MUSCLE algorithm was used to perform the MSA. The ESPript 3.0 Web site (https://espript.ibcp.fr/ESPript/ESPript/) was used to represent the sequence alignment.^68^ The secondary structure prediction was performed using JPRED web server (https://www.compbio.dundee.ac.uk/jpred/) using the default parameters.^69^

## 3 Results and Discussion

### 3.1 Structural Evaluation of HuR-mRNA and HuR Models

The domain architecture of HuR in its unbound state reveals a distinct spatial arrangement of its three RRMs, each adopting a characteristic fold, as described below. In the model (Figure 1B), the RRM1 domain (shown in blue) is located at the N-terminal region and adopts a βαββα fold, including an antiparallel β-sheet serving as the primary RNA-binding interface. RRM2 (indicated in red) is positioned in the middle and shows a structural organization similar to that of RRM1. RRM3 (shown in green), located in the C-terminal region, is separated from the other two RRMs through a flexible, unstructured hinge region (in gray) and adopts a βαββα fold. The spatial arrangement of these domains is consistent with prior structural and functional studies,^17–19^ supporting the hypothesis of a cooperative mechanism between the RRMs in RNA recognition and stabilization.^18^ In the bound HuR (Figure 1C), the structural arrangement of the RRMs becomes compact due to the presence of mRNA. The mRNA fragment is correctly positioned in the pocket between RRM1 (blue) and RRM2 (red) and complexes with the hinge region between RRM2 and RRM3 (gray). RRM1 assumes a structure that can be described as an βαβββα fold, while RRM2 takes on a structure that can be described as an βαββαβ fold. RRM3, on the other hand, adopts a different aβαβαβ fold. The initial models were also further validated through the ERRAT, VERIFY, WHATCHECK, and PROCHECK programs. The ERRAT analysis retrieved overall quality factors of 84.61 and 85.55% for unbound and bound HuR, respectively. The VERIFY program passed, with 70.82% of residues having an average 3D–1D score ≥0.1. The WHATCHECK and PROCHECK programs resulted in some warnings concerning bond lengths, angles, torsion angles, and side chain planarity deviations for both models, which have been fixed through MD simulations. The QMEANDisCo analysis retrieved reliable scores of 0.62 ± 0.05 and 0.61 ± 0.05 for the unbound and mRNA-bound HuR models, respectively. These intermediate scores are primarily attributed to the presence of unstructured regions, including the N- and C-termini as well as the RRM2–RRM3 hinge. Nonetheless, the core regions of the models, especially those critical for HuR-RNA recognition, consistently show higher local confidence scores, reinforcing the reliability of these structured interfaces (Supporting Information Figure S1). Furthermore, the sampling approach mitigates uncertainties in these low-confidence regions by identifying consensus conformations that recur across multiple independent predictions.

### 3.2 Analysis of the HuR-mRNA and HuR Atomistic Simulations

The structural stability of HuR in both its bound and unbound states was assessed by analyzing the Root-Mean-Square Deviation (RMSD) of backbone atoms (excluding hydrogens) throughout the MD simulations, using the initial frame as a reference. The RMSD profiles revealed an initial increase during the first 100 ns of the simulation, indicative of a routine structural reorganization occurring in the equilibration phase. Following this initial adjustment, the RMSD values exhibited oscillations around a plateau, as shown in Supporting Information Figure S2. This analysis determined the trajectory between 100 and 500 ns to represent the optimal convergence interval for subsequent analyses. Residue-specific flexibility was assessed by calculating the RMSF of the protein’s Cα atoms across the simulated trajectories (Figure 2, upper panel). As anticipated, the unbound HuR systems (Figure 2A) exhibited higher RMSF values than did the HuR-mRNA complexes (Figure 2B). Notably, the RMSF values corresponding to unstructured regions were consistently elevated in the unbound HuR systems relative to the HuR-mRNA complexes. GR analysis was carried out to evaluate the protein structures’ compactness. GR measures the overall compactness, quantifying the distribution of atoms around the protein’s center of mass. The results reveal that in all replicas, the unbound HuR (Figure 2C) protein displays higher GR values than HuR in complex with mRNA (Figure 2D), demonstrating a considerable increase in compactness upon mRNA binding. Although replica 2 of the HuR-mRNA complex shows a marginally higher GR within the complex group, it still remains more compact than the corresponding HuR-only replica.

**Figure 2:**
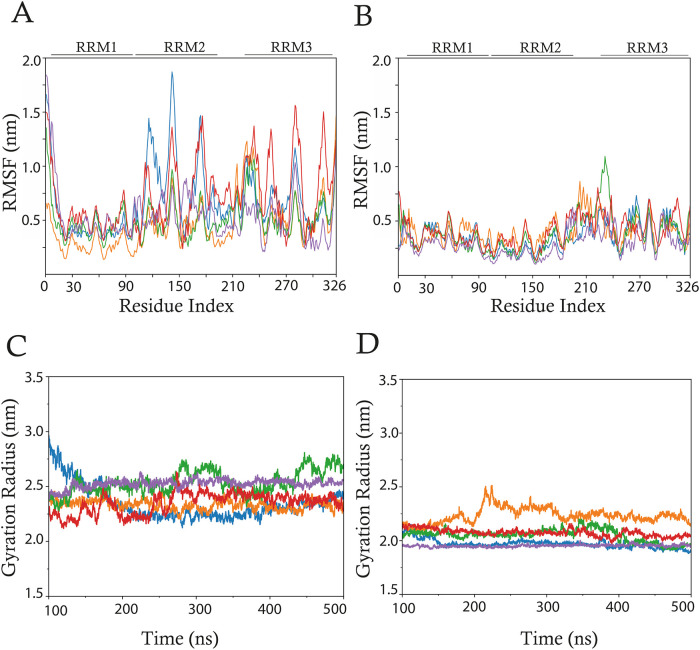
Structural analyses of unbound HuR and HuR-mRNA systems. Upper panels: RMSF calculated for the 326 Cα atoms of HuR of (A) unbound HuR and (B) HuR-mRNA systems for replicas 1 (blue), 2 (orange), 3 (green), 4 (red), and 5 (purple). Lower panels: Gyration radius calculated on all the protein heavy atoms of (C) HuR and (D) HuR-mRNA systems using the same color scheme for the different replicas.

We analyzed the interdomain geometry for both the unbound and mRNA-bound HuR systems by measuring the distances and angles between RRM pairs (Supporting Information Figure S3). In the absence of RNA, the RRM1–RRM2 distance is relatively stable, consistent with a semirigid arrangement that aligns with their principal function in RNA engagement.^4–6^ In contrast, distances involving the RRM3, particularly the RRM2–RRM3 distance, exhibited significant variability, reflecting an intrinsic flexibility that likely facilitates domain reorientation and protein dimerization^18^ (Supporting Information Figure S3A). Upon mRNA binding, these interdomain fluctuations were markedly dampened, especially between RRM2 and RRM3, indicating that the RNA ligand promotes a more compact and stable tertiary structure (Supporting Information Figure S3B).

To complement the distance-based analysis, a difference emerged when comparing inter-COM angle distributions between the unbound and mRNA-bound systems. The analysis revealed significant differences in its conformational dynamics. As shown in Supporting Information Figure S3C, the unbound protein exhibits a distribution characterized by three distinct peaks, indicating a high degree of flexibility and the ability to adopt multiple preferred conformations. In contrast, the binding of mRNA, as depicted in Supporting Information Figure S3D, results in a notable shift and a more clustered distribution of angles. This change suggests that mRNA binding induces a conformational change, leading to a more rigid and less dynamic structure. This rigidification of the protein is likely critical for its function in recognizing, binding, and regulating target mRNA molecules. Overall, while the unbound system maintains a broader conformational landscape, RNA binding restricts flexibility and enforces specific interdomain geometries. Cluster analysis on MD trajectories was also performed to identify prevalent conformational states adopted by HuR, using a 0.60 nm cutoff for cluster assignment based on structural similarity. This analysis revealed distinct conformational preferences between bound and unbound HuR. Unbound HuR predominantly sampled a more open conformation, while mRNA-bound HuR adopted a significantly more constrained structure characterized by a tight association of HuR with the mRNA. As evidenced by the reduced protein flexibility, these results also indicate that mRNA binding induces a conformational stabilization of HuR. This stabilization likely facilitates RRM3 domain interaction by positioning the protein in a more favorable orientation for dimerization. Furthermore, the decreased flexibility associated with mRNA binding may lower the entropic barrier to RRM3 association, promoting dimerization as a downstream consequence of mRNA engagement.

### 3.3 Validation of the Unbound HuR Structure through CG Simulations

While all-atom (AA) models offer the highest level of detail, their application is constrained by computational limitations when exploring processes exceeding microsecond time scales or involving complex systems such as large-scale motions.^37^ Consequently, coarse-grained (CG) models have emerged as a powerful alternative, enabling significant acceleration by reducing the system’s degrees of freedom.^37^ Among these, the Martini force field is widely recognized as a leading CG model. The recently released Martini 3.0,^37^ with its refined interaction matrix and expanded bead-type library, promises to enhance the accuracy of protein topologies, thereby facilitating more realistic simulations of complex phenomena. To further validate the HuR structure predicted by RoseTTAFoldNA and to support the results obtained from AA simulations, we conducted CG-MD simulations. Starting from a linearized model of HuR, where RRM domain structures were maintained but interdomain interactions were removed, we expected to observe spontaneous association toward the predicted assembled structure. This approach excluded potential artifacts arising from limitations inherent in both predictive and atomistic methodologies. CG simulations were performed by using the HuR protein structure derived from the centroid of the fifth AA replica after removing the mRNA fragment. The conversion from the AA to CG representation is shown in Figure 3A. Visual inspection of the CG trajectories revealed a transition from extended to compact state within a 10.0 μs simulation time frame, consistent with the HuR assembly predicted by RoseTTAFoldNA. To quantify the observed conformational transition, RMSD analysis was performed using the centroid structure of each replica (Figure 3C) as a reference frame (Supporting Information Figure S4A). This analysis demonstrated a significant decrease in RMSD values in all replicas, indicating convergence toward a compact structural assembly. This reduction was particularly evident in replicas 1, 3, and 5, suggesting a more efficient packing process in these simulations. Additionally, RMSF analysis (Supporting Information Figure S4B) confirmed the enhanced flexibility of the RRM2–RRM3 linker and RRM3 domain, highlighting their bendable nature. The GR was also evaluated to confirm the protein’s compactness, employing the centroid structure of each replica as a reference frame (Figure 3B). The GR analysis revealed a progressive increase in compactness, indicated by a decrease in the GR values over time. Specifically, the GR converged to a stable average value of approximately 2.0 nm during the final 5 μs of the simulation, implying the attainment of a compact folded state. This observation is supported by the GR analysis performed on AA simulations of the same protein (Figure 2B).

**Figure 3:**
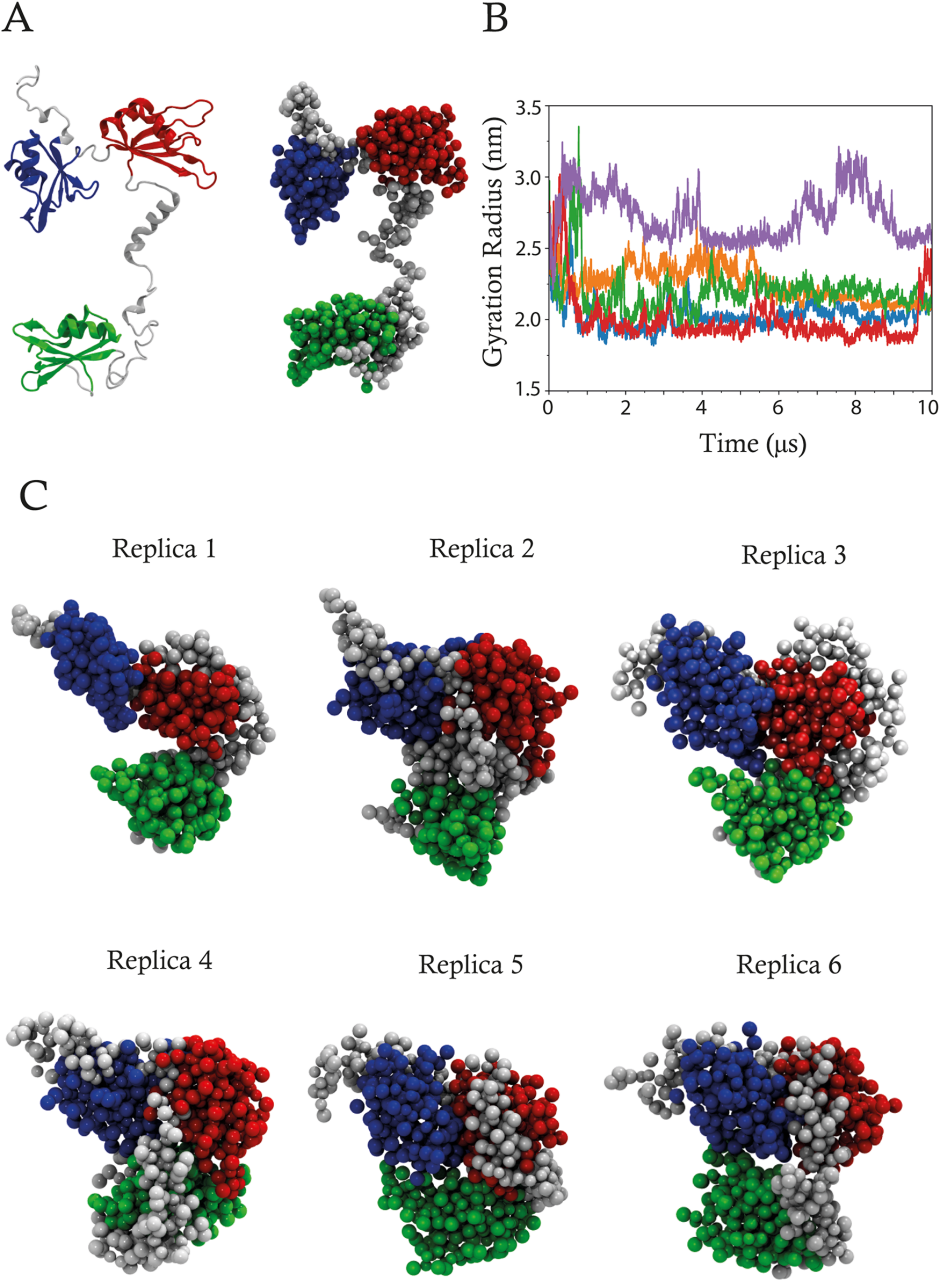
Coarse-grained (CG) modeling of HuR. The three RNA-recognition motifs (RRMs) are distinguished by color: RRM1 (blue), RRM2 (red), and RRM3 (green). (A) Cartoon representation of the protein model, obtained from atomistic MD simulations of the HuR-mRNA complex and linearized using PyMOL and Chimera, is shown (left panel), alongside the bead representation of the CG model (right panel). (B) Gyration radius of replica 1 (blue), 2 (orange), 3 (green), 4 (red), and 5 (purple). (C) Bead representation of centroids, extracted from the clustering analysis of the CG simulations, is shown.

The interdomain geometry of unbound HuR also for the coarse-grained (CG) simulations (Supporting Information Figure S5) was analyzed. The CG distance analysis confirmed the AA findings, with the RRM1–RRM2 pair maintaining close interaction, while the RRM3 domain samples a broad conformational landscape, underscoring its high intrinsic flexibility (Supporting Information Figure S5A). Consistently, the interdomain COM angle distribution of the CG system was broad (Supporting Information Figure S5B), indicating that, without additional constraints, the relative orientations of the three RRM domains are highly flexible and largely unrestrained. This diffuse distribution reflects the high flexibility of the RRM domains, particularly RRM3. It suggests a lack of stable interdomain arrangements, in accordance with a conformationally heterogeneous ensemble in which multiple geometries are accessible without a dominant population. Collectively, these CG results strongly support the conclusions drawn from the AA data regarding the dynamic character of unbound HuR, particularly the combination of a stable RRM1–RRM2 interface with the pronounced flexibility involving RRM3. The successful convergence of the CG simulations toward the predicted assemblies and the data arising from AA simulations, despite initiating from a non-native conformation, underscores the reliability of our findings and provides independent confirmation of the HuR structural model.

### 3.4 Principal Component Analysis of HuR-mRNA and HuR AA Simulations

To evaluate the dominant collective motions of unbound HuR and the HuR-mRNA complex, principal component analysis (PCA) was performed on concatenated MD trajectories, each 2.5 μs in length, effectively increasing the total conformational sampling for the analysis of protein dynamics. PCA was conducted using the Cα atoms of the 326 protein residues, and the first two principal components (PC1 and PC2), representing the highest variance in data, were visualized as hexbin plots (Figure 4). The two-dimensional (2D) projection of the unbound HuR system (Figure 4A) revealed a broad, dispersed distribution, indicating the sampling of a larger conformational space compared with the HuR-mRNA complex (Figure 4B), which exhibited a more compact distribution. This again confirms that HuR samples a restricted conformational space upon mRNA binding, consistent with a stabilizing effect that may enhance specific mRNA interactions by reducing HuR’s structural flexibility. The dynamic behavior of the RRM2–RRM3 linker and RRM3 domain (residues 187–322) was also investigated by generating DCCMs from covariance matrices. This approach allows the analysis of correlated motions between residue pairs in HuR, comparing mRNA’s absence (Figure 5A) and presence (Figure 5B). In DCCMs, positive values represent residues moving in the same direction (correlated motion), while negative values represent movement in opposite directions (anticorrelated motion). Upon ligand binding, we observed moderate yet significant changes in these motions, primarily in the RRM3 domain and its adjacent linker region (residues 230–322) (Figure 5B). In striking contrast, the dynamic patterns within the dimerization interface (residues 190–227) remained highly similar to those of the unbound state. This remarkable conservation suggests that the intrinsic flexibility required for dimerization is preserved when HuR engages RNA. Consequently, the protein remains structurally ready for self-association regardless of its RNA-binding status, a finding consistent with studies showing dimerization can occur both before and after ligand engagement.^17–19^ Therefore, this DCCM analysis not only clarifies the functional interplay between RNA binding and dimerization but also validates our HuR-mRNA model by demonstrating that it accurately captures this functionally critical dynamic behavior.

**Figure 4:**
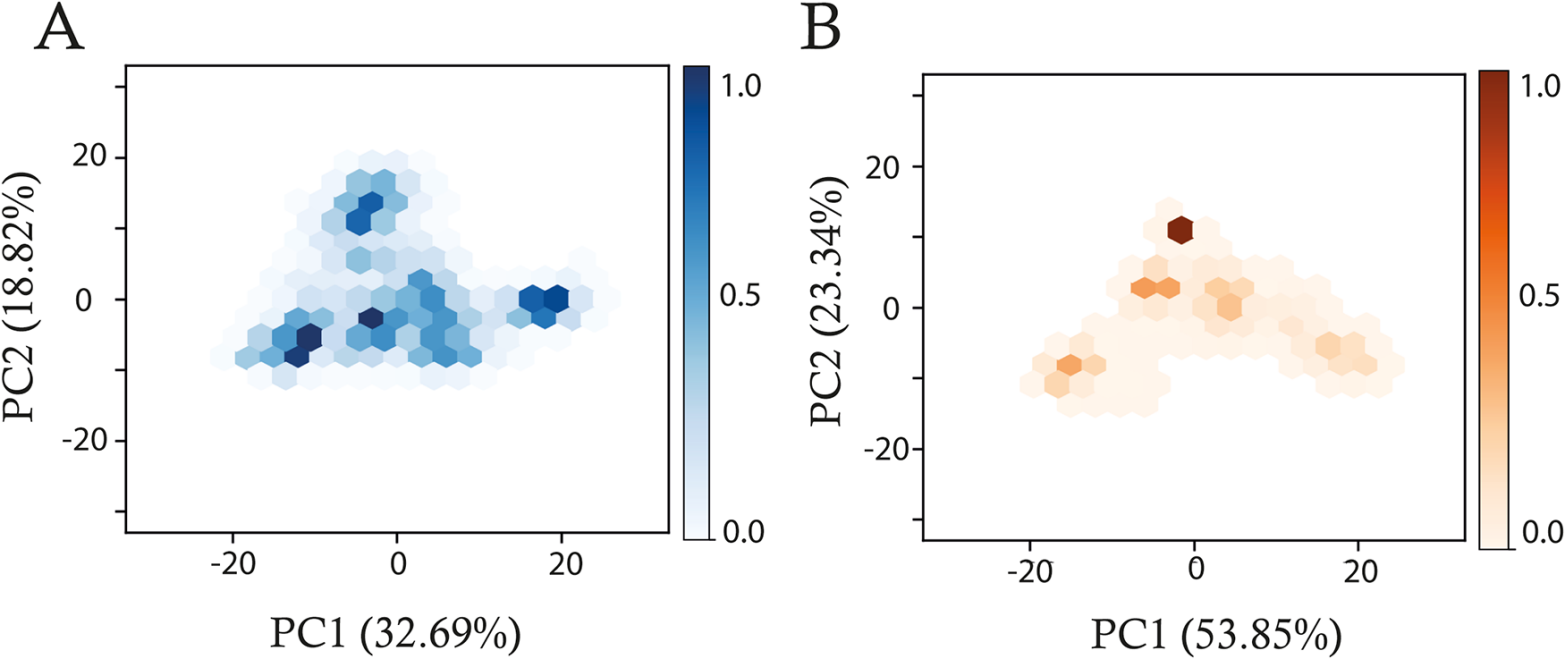
Hexbin plots depicting the 2D projections of principal component 1 (PC1) and principal component 2 (PC2) for concatenated trajectories of (A) unbound HuR and (B) HuR-mRNA systems. The percentage variance captured by each principal component is indicated. Each hexagon represents the density of sampled conformational states, with color intensity reflecting the logarithmic density of configurations. The color bars indicate normalized density values ranging from 0 (white) to 1 (dark color).

**Figure 5:**
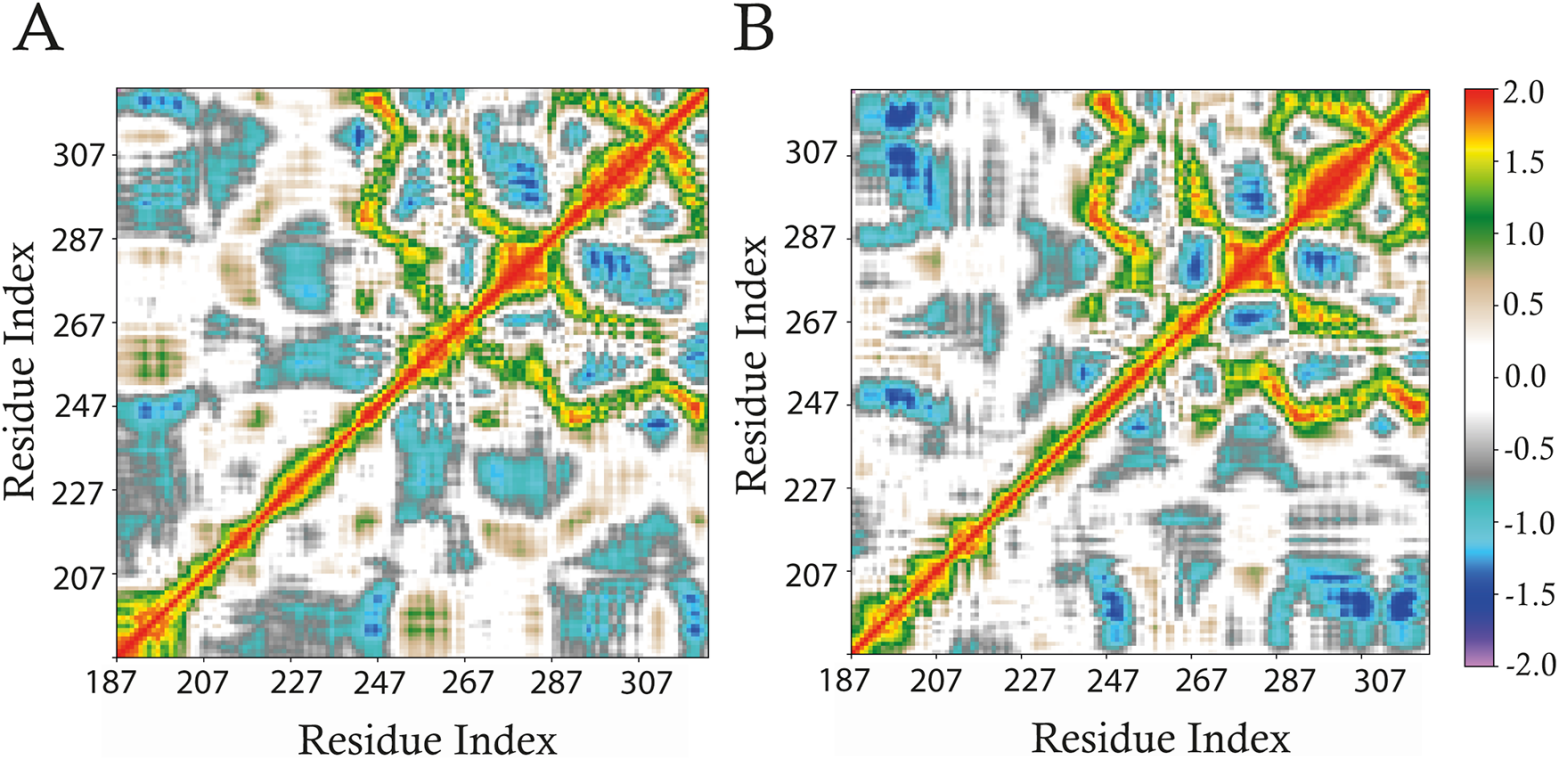
Dynamic cross-correlation matrices (DCCM) calculated on the 126 Cα atoms of the RRM2–RRM3 linker and RRM3 region (residues 187–322) of (A) unbound HuR, and (B) HuR-mRNA systems. Color coding is reported in the figure legend. Positive values between two residues indicate a correlated motion, meaning that the residues are moving in the same direction, while negative values indicate an anticorrelated motion, meaning that the residues are moving in different directions.

### 3.5 Interaction Analysis of HuR-mRNA and HuR Systems

To characterize the interactions between HuR and mRNA, as well as HuR intramolecular interactions, hydrogen bond (HB) networks, contact frequencies, and π–π stacking interactions, have been analyzed. A 20% occupancy threshold was used to identify transient interactions, such as H-bonds, which are known to form and break frequently during MD simulations. In fact, while these transient interactions may not always be present, they could potentially play a functionally relevant role. This analysis revealed comparable numbers of HBs in both unbound HuR and HuR-mRNA systems (Supporting Information Figure S6), suggesting structural stability within the RRM domains and validating the predicted model. Notably, stable HBs were observed between the RRM2–RRM3 linker and the RRM3 domain as well as between HuR and RNA (Supporting Information Figure S7). Further examination of the involved residues (Supporting Information Figure S8) highlighted the substantial participation of K285, located within the RNP1 motif of RRM3, and of K320, making HBs across four of the five replicas. Contact frequency analysis between the RRM2–RRM3 linker and the RRM3 domain, as well as between HuR and RNA, was performed using a value of 50% for the persistence threshold to ensure that the identified contacts are not randomly occurring but represent recurrent features throughout the simulations (Supporting Information Figure S9). The results further emphasize the stabilizing role of K285. Additional residues, including P187, N192, H212, H213, Q216, R217, K274, K320, and N322, consistently form contacts with RNA, indicating their valuable contribution to stabilizing the protein–RNA interface. Moreover, F287 (RNP1) and Y249 (RNP2) demonstrated persistent contact in four of the five MD simulations. π–π stacking interactions between aromatic residues of HuR and RNA bases were analyzed by using a value of 40% for the occupancy cutoff, which allowed the identification of relevant stacking interactions without excluding those that, despite variability, may still play a functional role. These interactions are crucial in protein–RNA complexes, particularly for RRMs, where aromatic side chains stabilize nucleobases through planar stacking. In HuR, such interactions enhance RNA affinity, orientation, and complex stability, supporting the model reliability. Three pairs, namely, Y26–U22, Y5–U22, and F218–U12, met the interaction criteria (Figure 6). Among these, the Y26–U22 pair was consistently observed in all replicas, suggesting a critical role for Y26 in the mRNA binding. To support this observation, minimum distance analyses between the geometric center of the aromatic residues and the RNA bases were performed, as discussed in Section 2. The Y26–U22 pair exhibited stable distances in four replicas with minor fluctuations observed only in the final 100 ns of replica 1 (Figure 6A). In contrast, the Y5–U22 and F218–U12 pairs displayed higher minimum distances, with an average value of 2.4 ± 0.7 nm, indicating the transient interruption of the interactions (Figure 6A). These findings show that Y26 is the primary contributor to stable π–π interactions, as evidenced by its persistent stacking across all five replicas (Figure 6B). In contrast, Y5 and F218 appear to play more transient roles, likely involved in the initial recognition of the mRNA. Finally, to estimate the binding affinity between the protein and RNA, molecular mechanics/Poisson–Boltzmann surface area (MM/PBSA) calculations^55^ were performed, indicating similar interaction energy in all simulation replicas (Supporting Information Figure S10). Notably, the electrostatic component contributes significantly to the overall binding energy, highlighting its dominant role in stabilizing the complex. This consistency across replicas suggests a high degree of reliability in the model, further validating the simulation setup and the predicted protein–RNA-binding affinity.

**Figure 6:**
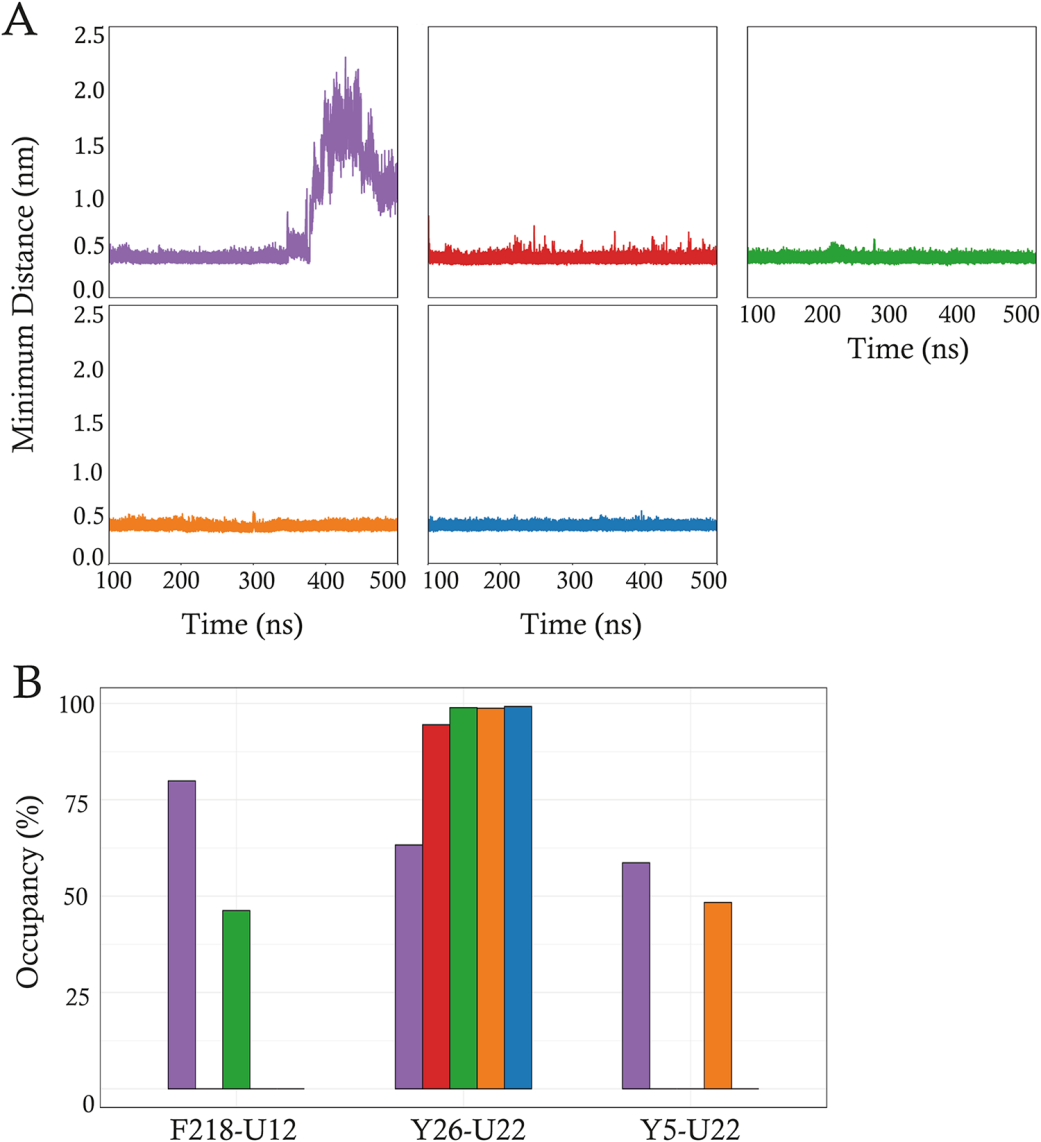
Summary of the π–π stacking pairs statistics. (A) Minimum distance analysis of the Y26–U22 π–π stacking pair for replica 1 (violet), 2 (red), 3 (green), 4 (orange), and 5 (blue). For each plot, the average value and standard deviation (average ± standard deviation) are reported. (B) Barplot of the time occupancy for each found pair. Colors follow the same scheme as in panel (A).

### 3.6 Y26 Residue Conservation

The ConSurf web server was used to evaluate the evolutionary conservation of the Y26 residue within the HuR protein. This tool assesses conservation based on phylogenetic relationships among homologous sequences. Since the degree of evolutionary conservation at an amino acid position often reflects its structural and functional importance, this analysis can provide insights into the Y26 role. The results obtained were mapped to the HuR protein structure derived from the HuR-mRNA complex after the removal of the mRNA fragment. As shown in Figure 7, Y26 exhibits significant evolutionary conservation. At this position, tyrosine is conserved in 98% of the analyzed sequences, while cysteine and phenylalanine appear with a frequency below 1% (detailed rates are in Supporting Information File S1). HuR belongs to the ELAV/Hu family of RBPs, which also includes HuB, HuC, and HuD in mammals. These proteins share a high degree of sequence similarity, particularly within their RRMs.^3,9^ Available Hu protein sequences belonging to structures retrieved from the PDB have been aligned to investigate structural conservation relevant to HuR. Currently, five partial structures of ELAV family members are deposited in the PDB: three *M. musculus* HuC fragments (PDB IDs: 1D8Z, 1D9A, 1FNX) and two *H. sapiens* HuD fragments (PDB IDs: 1FXL, 1G2E). No structures were found for HuB. These protein sequences were aligned using the MUSCLE algorithm (Supporting Information Figure S11). Multiple alignments revealed that tyrosine is conserved at the position corresponding to Y26 in HuR in four out of the five available structures. The high degree of evolutionary conservation observed for this residue suggests its involvement in crucial physiological functions such as RNA recognition. This is further supported by its location within the RNP2 motif of RRM1, which is a domain essential for recognizing and binding AREs in target RNAs. Y26 has been reported to be part of an aromatic cage, along with Y63 and F151, which plays a critical role in positioning the inhibitor TM7nox.^70^ This structural arrangement implies a strong potential contribution of Y26, either directly or indirectly, to interactions with RNA. Supporting the role of tyrosines, previous mutagenesis studies on Y109 demonstrated that a mutation in this position significantly reduces RNA-binding affinity.^71^ This finding is consistent with observations for an equivalent residue, Y135, in the protein HuD.^66^ Predictions were made using the JPRED web server to explore whether the conservation of Y26 impacts the local secondary structure (Supporting Information Figure S12). These predictions indicated that the secondary structural context surrounding the Y26 position is consistently maintained across the aligned sequences, suggesting that residue conservation may be essential for preserving the local fold. To date, only three specific mutations in HuR (S138A, S221A, and S318A)^72^ have been experimentally characterized. The absence of observed natural variants at position 26 in population databases, combined with its high evolutionary conservation, strongly suggests that mutations of this residue are likely detrimental, potentially disrupting critical protein–RNA interactions and leading to significant functional impairment or reduced organismal fitness.

**Figure 7:**
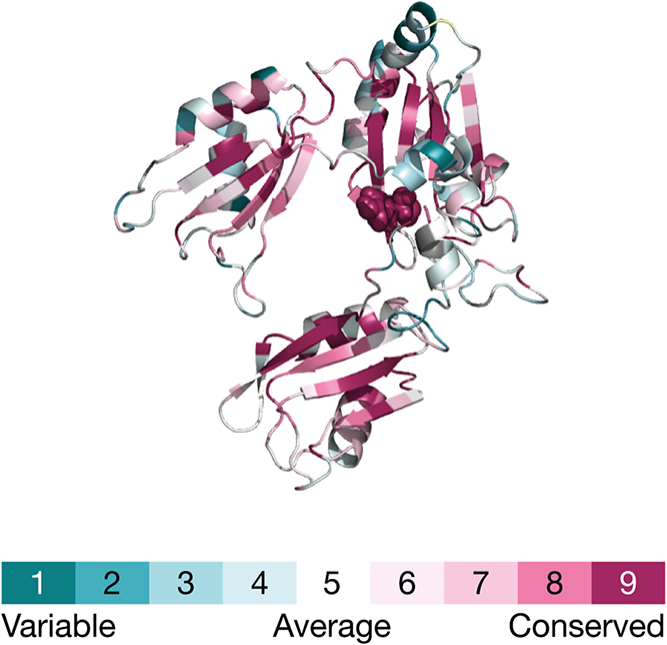
ConSurf web server prediction of the evolutionary conservation of HuR structure. The residue Y26 is highlighted with a spacefill representation. The color legend is provided, with a scale from cyan, indicating variable residues, to magenta, indicating highly conserved residues.

### 3.7 Analysis of the Mutant Y26A Atomistic Simulations

To investigate the functional relevance of residue Y26, the Y26A HuR mutant was modeled and simulated through classical molecular dynamics. Structural stability was first assessed through RMSD analysis (Supporting Information Figure S13A), which confirmed stable behavior across all replicas, thus validating the use of the 100–500 ns time window for comparison with the wild-type (WT) system. The RMSF analysis (Figure 8A) revealed a global increase in flexibility across all three RNA-recognition motifs (RRMs) in the Y26A mutant compared with the WT (Figure 2B). This enhanced mobility originates in RRM1, where the mutation is located, and subsequently propagates to RRM2 and RRM3. Such an effect suggests a long-range destabilization that alters the dynamic equilibrium of the entire protein. These findings are supported by interdomain distance analyses (Supporting Information Figure S13C), which show a substantial increase in the RRM1–RRM2 distance in Y26A compared to the WT (Supporting Information Figure S3B). In contrast to the WT system (Supporting Information Figure S3C), which displayed multiple distinct interdomain COM angle populations, the Y26A mutant collapsed into a much narrower distribution centered around ∼70° (Supporting Information Figure S13D). This reduction in conformational diversity suggests that the Y26A mutation severely restricts the dynamic sampling of interdomain orientations, effectively locking the RRM domains into a single predominant geometry. While RNA binding generally increases conformational specificity, yet still allows multiple favored states, the Y26A mutation prevents exploration of the broader conformational landscape, suggesting a potential loss of functional flexibility across the protein. A moderate increase in the GR (Figure 8B) is observed from 300 ns onward in three of five replicas, further suggesting that the Y26A mutation promotes an overall expansion of the protein’s structure. This effect is likely to result from weakened interdomain interactions and reduced packing efficiency. To compare the conformational dynamics of the Y26A mutant with those of the WT, we performed principal component analysis (PCA) on the 126 Cα atoms of the RRM2 and RRM3 domains from the concatenated MD trajectories. The analysis revealed an expanded conformational landscape for the Y26A mutant (Figure 8C), indicating a broader sampling of dynamic states. This finding was corroborated by dynamic cross-correlation matrix (DCCM) analysis, which showed a general weakening of both correlated and anticorrelated motions in the mutant (Supporting Information Figure S13B) relative to the WT (Figure 5B). This alteration in correlated motions suggests an impaired dynamic coupling between structural elements, which may compromise the protein’s function. Interaction analyses support the structural destabilization hypothesis. The Y26A substitution results in the loss of the key π–π stacking interaction between the aromatic ring of tyrosine and the RNA. Although the total number of intramolecular hydrogen bonds remains comparable to the WT system (Supporting Information Figure S13E vs Figure S6B), the number of persistent hydrogen bonds between the RRM2–RRM3 linker, RRM3, and the RNA, is slightly reduced in Y26A (Figure 8D). Hydrogen bonds were filtered using a 20% occupancy threshold, and results (Supporting Information Figure S13F) reveal a loss of stable contacts in the mutant, which may further weaken the interaction with the RNA molecule. The interaction energy between HuR and the RNA was also evaluated *via* MM/PBSA calculations. The binding energy components of the Y26A mutant are comparable to those calculated for the WT (Figures S13G and S10), suggesting the mutation does not drastically impair binding affinity. Instead, its primary effects are on the conformational stability and interdomain coordination, probably required for optimal protein function. In summary, while the Y26A mutation preserves RNA-binding affinity, it induces a globally destabilized structure characterized by increased flexibility, altered interdomain organization, and weakened dynamic coupling. These findings thus underscore the critical role of Y26 in maintaining the overall structural and functional integrity of HuR.

**Figure 8:**
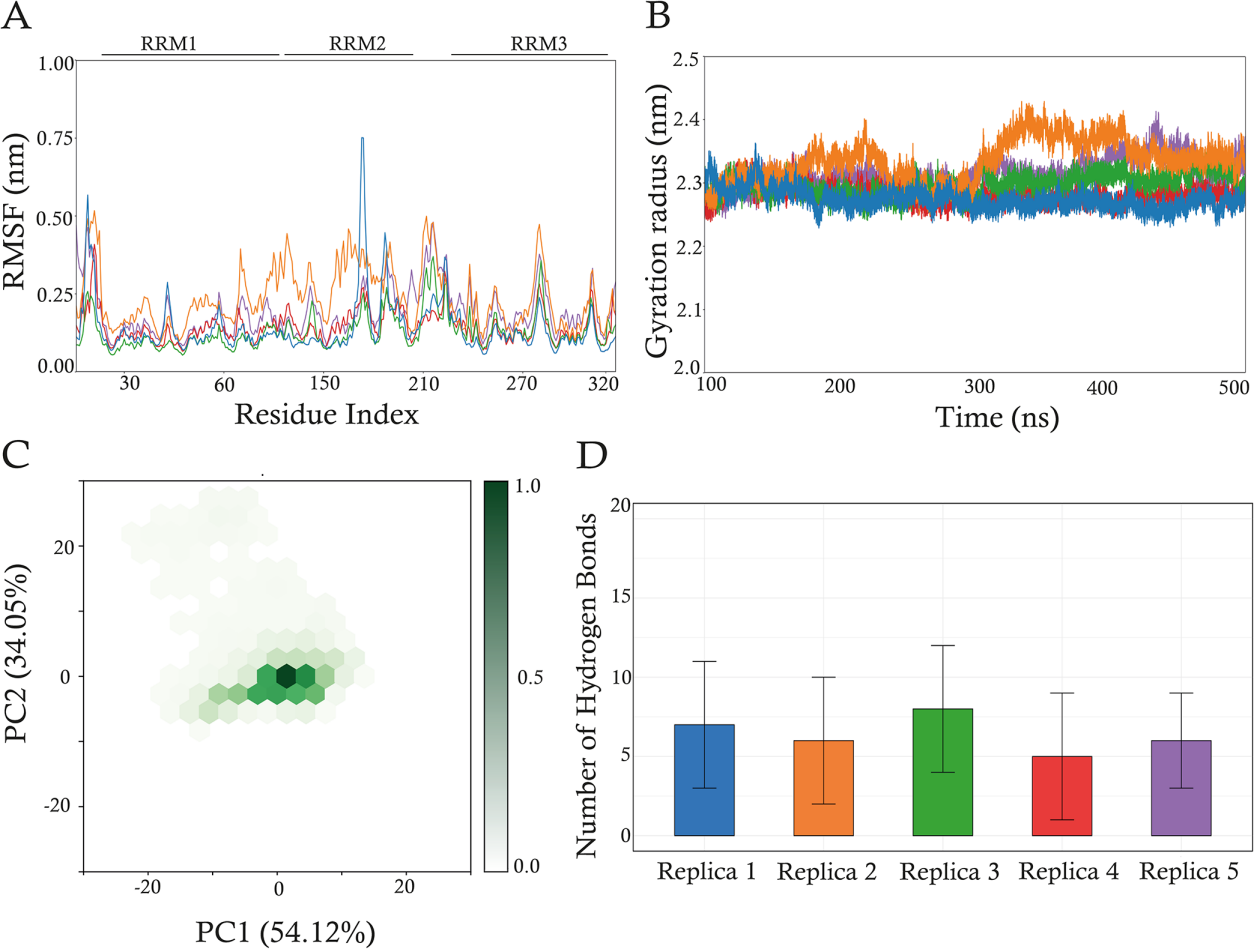
Structural analyses of the Y26A mutant system. (A) RMSF calculated for the 326 Cα atoms of the Y26A system for the five simulation replicas. (B) Gyration radius calculated on all the protein heavy atoms of Y26A systems. Colors are assigned according to the scheme reported in Figure 2. (C) Hexbin plots depicting the 2D projections of principal component 1 (PC1) and principal component 2 (PC2) for concatenated trajectories of Y26A systems. The percentage of variance captured by each principal component is indicated. Each hexagon represents the density of sampled conformational states, with the color bars indicating normalized density values ranging from 0 (white) to 1 (dark color). (D) Number of hydrogen bonds, expressed as average ± standard deviation, between the RRM2–RRM3 linker and RRM3 domain region and the RNA of Y26A systems.

## 4 Conclusions

The critical role of HuR in regulating different biological pathways, including cellular stress responses and cancer progression, is well-established, with its overexpression correlating with disease severity in numerous cancers such as lung, breast, liver, and colon.^22–30^ To address the lack of an experimentally validated 3D structure for full-length HuR, we developed an *in silico* model of HuR, both alone and in complex with mRNA, using RoseTTAFoldNA.^34^ Both systems were subjected to five independent replicas of classical MD simulations, each for 500 ns, to assess the structural integrity and behavior of the models. The analysis of RMSF (Figure 2A,B) and GR (Figure 2C,D) revealed that the HuR-mRNA complexes are characterized by a significant reduction in atomic flexibility and an increase in compactness compared to the unbound HuR. This increased compactness suggests a more ordered and thermodynamically stable conformation, which is likely to facilitate intermolecular interactions and enhance the potential for HuR dimerization.^8^ Coarse-grained (CG) simulations further corroborated these atomistic findings by showing a consistent trend toward increased packing of the protein structure, even in the absence of RNA (Figure 3B). PCA analyses demonstrated that upon mRNA binding, HuR’s conformational exploration is confined, indicating a stabilizing effect that could enhance specific mRNA interactions by reducing structural variability (Figure 4). Interestingly, DCCM analysis (Figure 5) showed that mRNA binding induced only minimal changes in the correlated motions of the RRM2–RRM3 linker and RRM3 domain, with patterns closely resembling those observed in the unbound state. This suggests that HuR dimerization can occur both before and after mRNA binding, which is consistent with existing literature.^18^ Interaction network analyses of the HuR-mRNA models revealed a comparable number of intraprotein hydrogen bonds (HBs) across all simulations, supporting the reliability of the modeled RRMs (Supporting Information Figures S6 and S7). Stable interactions involving residues within the RNP1 and RNP2 motifs (Supporting Information Figure S8), critical for RNA recognition and stabilization,^17–19^ were identified. Specifically, residue K285, located within the RNP1 motif of the RRM3 domain, formed strong interactions with RNA, evidenced by the high contact frequency and occupancy rates in the simulation replicas. Other residues, including F287 (RNP1) and Y249 (RNP2), also formed persistent contacts in four of the five replicas. A cohort of additional residues (P187, N192, H212, H213, Q216, R217, K274, K320, and N322) were also identified as potentially contributing to RNA stabilization after HuR recognition. A key structural insight was the identification of a significant π–π stacking interaction between Y26 and U22, which was characterized by high occupancy (Figure 6). Evolutionary analysis and protein sequence alignment show that Y26 in the RRM1 region is highly conserved across species, including *H. sapiens* and *M. musculus*. This finding, based on phylogenetic relationships (Figure 7) and supported by analyses of other ELAV/Hu family proteins like HuC and HuD (Supporting Information Figure S11), emphasizes the functional and evolutionary importance of this amino acid. Notably, no natural variants or mutations have been reported at this position to date,^72^ suggesting that Y26 is not only essential for binding stability but also for preserving the structural integrity and functional competence of HuR. To validate the role of Y26, we simulated and analyzed the Y26A mutation, which showed a destabilizing effect on the protein structure. The mutation resulted in increased RMSF and GR (Figure 8A,B, respectively), indicating a more flexible and less compact structure that samples a broader conformational space (Figure 8C). These findings are supported by a reduction in the HB network among the RRM2–RRM3 linker, the RRM3 domain, and RNA bases (Figures 8D and S13F). Additionally, the DCCM of the mutant showed a loss of correlated motions, suggesting altered interdomain communication (Supporting Information Figure S13B). These results underscore the functional significance of Y26 and the consequences of its substitution, which are primarily due to the loss of the critical π–π stacking interaction. In conclusion, this study proposes a reliable computational model of the HuR-mRNA complex, yielding significant insights into the structural determinants of HuR’s RNA-binding mechanism. The model substantiates the critical role of Y26 in stabilizing the protein–RNA interface and elucidates the contributions of additional key residues within the RRM domains to HuR’s overall stability and mRNA binding affinity.

## Supplementary Material





## Data Availability

The starting unbound and mRNA-bound models predicted using RoseTTAFoldNA and the refined mRNA-bound HuR model presented in this study are openly available in ModelArchive database under accession ID 10.5452**/** *ma-cvndv*.

## Notes

Per-residue QMEANDisCo quality score of predicted models (Figure S1); root-mean-square deviations (RMSD) of HuR and HuR-mRNA complexes (Figure S2); interdomain analyses between HuR RRM domains over time in AA-MD trajectories (Figure S3); root-mean-square deviations and root-mean-square fluctuations on the CG-HuR MD trajectories (Figure S4); interdomain analyses between HuR RRM domains over time in CG-MD trajectories (Figure S5); intramolecular hydrogen bond network calculated for the HuR and HuR-mRNA systems (Figure S6); hydrogen bond network between the RRM2–RRM3 linker and RRM3 domain region and the RNA of HuR-mRNA systems (Figure S7); heatmap of the linker-RRM3 domain region and RNA hydrogen bond pairs (Figure S8); residues of the linker-RRM3 domain region contacting the RNA bases (Figure S9); MM/PBSA analyses of the MD trajectories of HuR-mRNA systems (Figure S10); protein sequence alignment of Hu family proteins (Figure S11); secondary structure and solvent accessibility predictions for the HuR protein sequence (Figure S12); and structural and interaction analyses of the Y26A mutant atomistic molecular dynamics (Figure S13) (PDF)
Conservation rate of Y26 retrieved from ConSurf web server prediction (File S1) (TXT)

## Abbreviations

RBP: RNA-binding protein
RRMs: RNA-recognition motifs
mRNA: mRNA
ELAV: embryonic lethal abnormal vision
UTR: untranslated region
AREs: AU-rich elements
RNP: ribonucleoprotein
HSN: nucleocytoplasmic shuttling sequence
PDB: Protein Data Bank
MD: molecular dynamics
AA: all-atom
CG: coarse grained
RMSD: root-mean-square deviation
RMSF: root-mean-square fluctuation
GR: gyration radius
DCCM: dynamic cross-correlation matrix
PCA: principal component analysis
HB: hydrogen bonds
MM/PBSA: molecular mechanics/Poisson–Boltzmann surface area
MSA: multiple sequence alignment
WT: wild-type
