# Supplementary material for: In Silico Structural Modeling of the HuR-mRNA Complex: Insights into Structural and Functional Regulation
Source: J Chem Inf Model. 2025 Oct 1;65(20):11158–72. doi: 10.1021/acs.jcim.5c01028 (PMC12570136; doi:10.1021/acs.jcim.5c01028)
Supplement: Supplementary file 1 [file ci5c01028_si_001.pdf]

In Silico Structural Modelling of HuR-mRNA Complex: Insights into Structural and Functional Regulation

Davide Pietrafesa [1], *Alice Romeo* [1], *Fabio Giovanni Tucci* [1], *Paola Fiorani* [1,2], *Federico Iacovelli* [1*] *and Mattia Falconi* [1*]

[1] Department of Biology, University of Rome "Tor Vergata", Via della Ricerca Scientifica 1, 00133 Rome, Italy

[2] Institute of Translational Pharmacology, National Research Council, CNR, Via del Fosso del Cavaliere 100, 00133 Rome, Italy

* Corresponding authors: federico.iacovelli@uniroma2.it, falconi@uniroma2.it

Supporting Materials

S1. Per-residue QMEANDisCo quality score of predicted models.

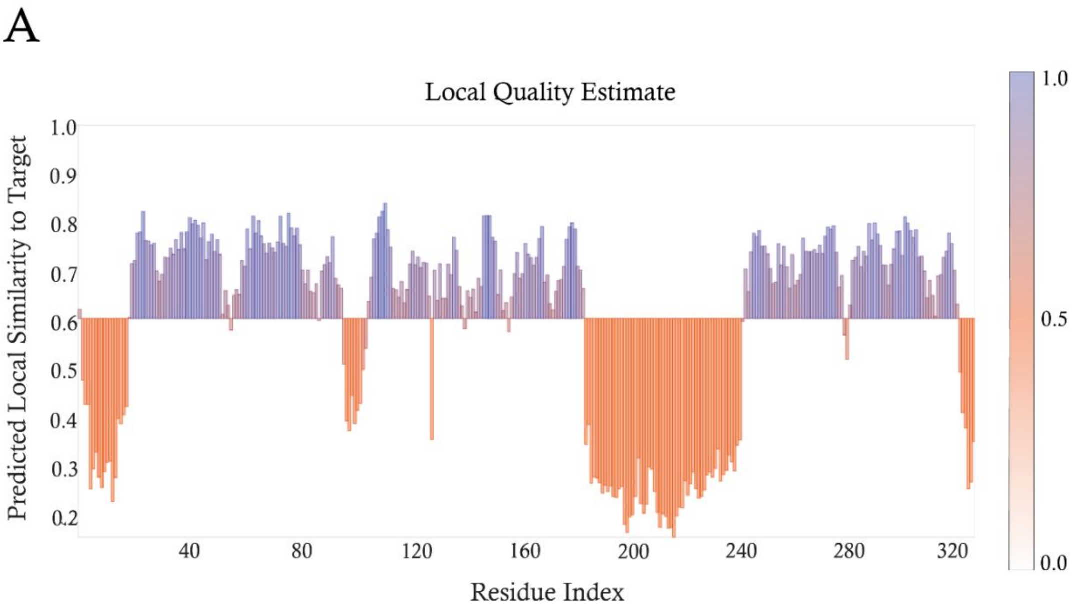

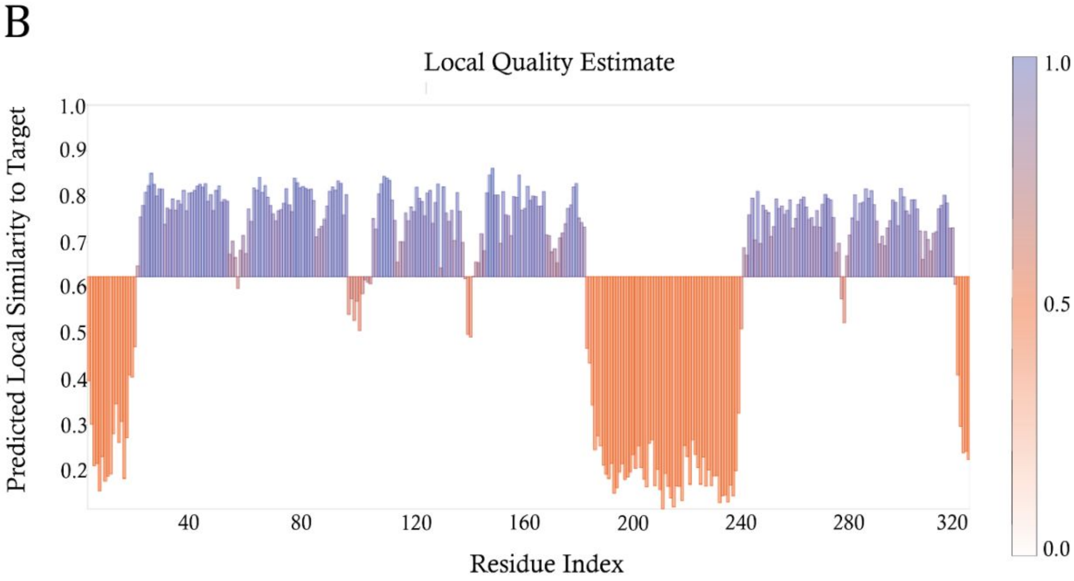

Figure S1. Per-residue QMEANDisCo quality score of (A) model 676 unbound-HuR complex and (B) model 838 HuR-mRNA complex. The color bar represents the local quality estimate, ranging from 0 (low predicted similarity to the target) to 1 (high predicted similarity to the target).

S2. Root Mean Square Deviations (RMSD) of HuR and HuR-mRNA complexes.

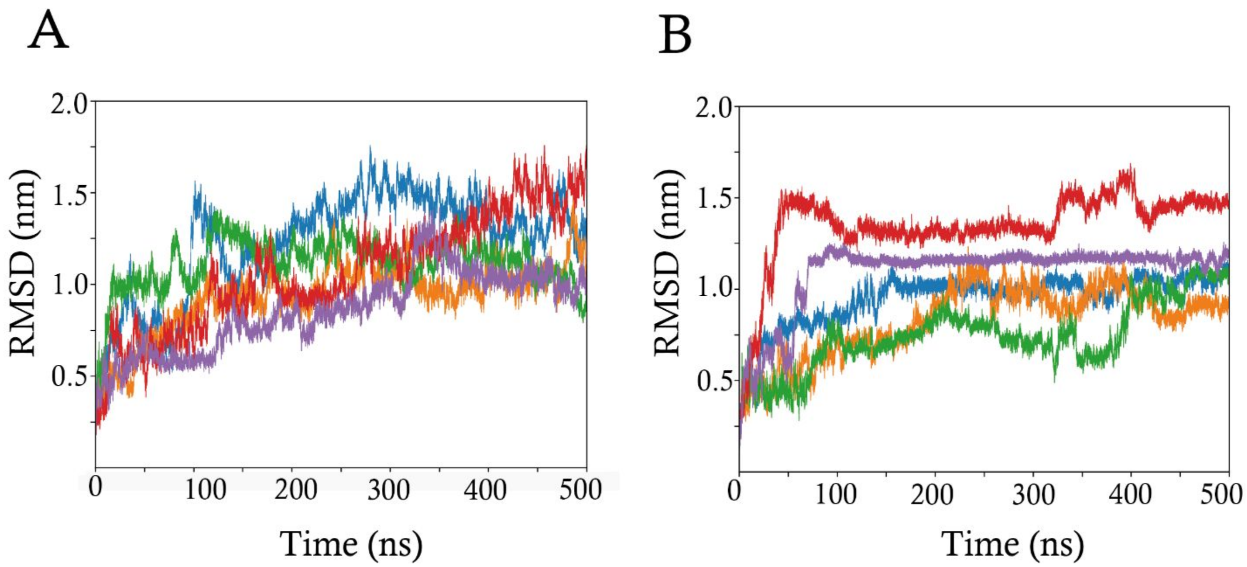

Figure S2. Root Mean Square Deviation (RMSD) of (A) HuR and (B) HuR-mRNA complexes for replicas 1 (blue), 2 (orange), 3 (green), 4 (red), and 5 (purple).

S3. Interdomains distance analyses between HuR RRM domains over time in AA-MD trajectories.

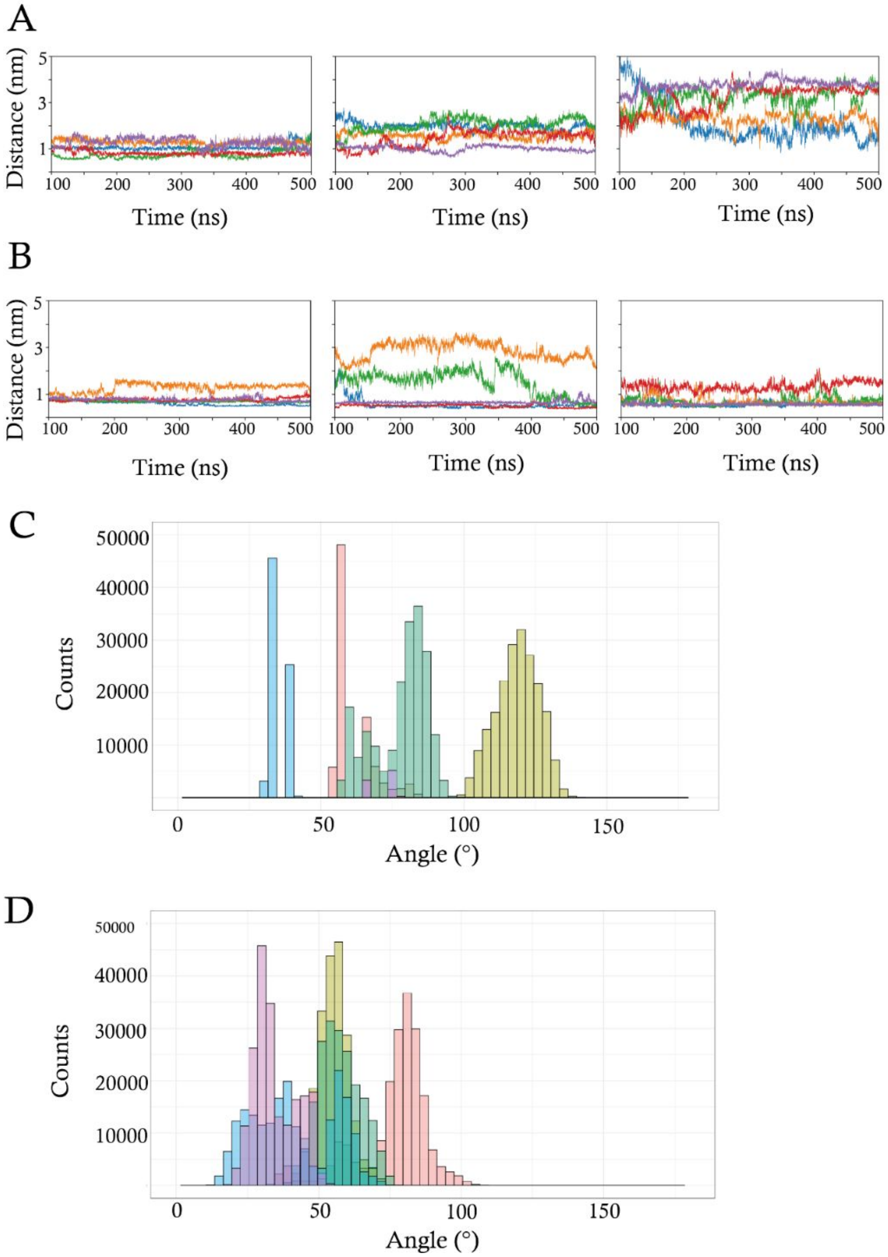

Figure S3. Center of Mass distances between interdomains RRM1-RRM2 (left), RRM1-RRM3 (center), and RRM2-RRM3 (right) of HuR unbound (A) and HuR-mRNA bound (B) for replicas 1 (blue), 2 (orange), 3 (green), 4 (red), and 5 (purple). The distances were calculated on all atoms of domains, excluding the hydrogens. Distribution of the angle, expressed in degrees, formed by

the centers of mass of RRM1, RRM2, and RRM3 domains of HuR unbound (C) and HuR-mRNA bound (D). Colors are assigned according to the scheme reported in Figure S2.

S4. Root Mean Square Deviations and Root Mean Square Fluctuations on the CG-HuR MD trajectories.

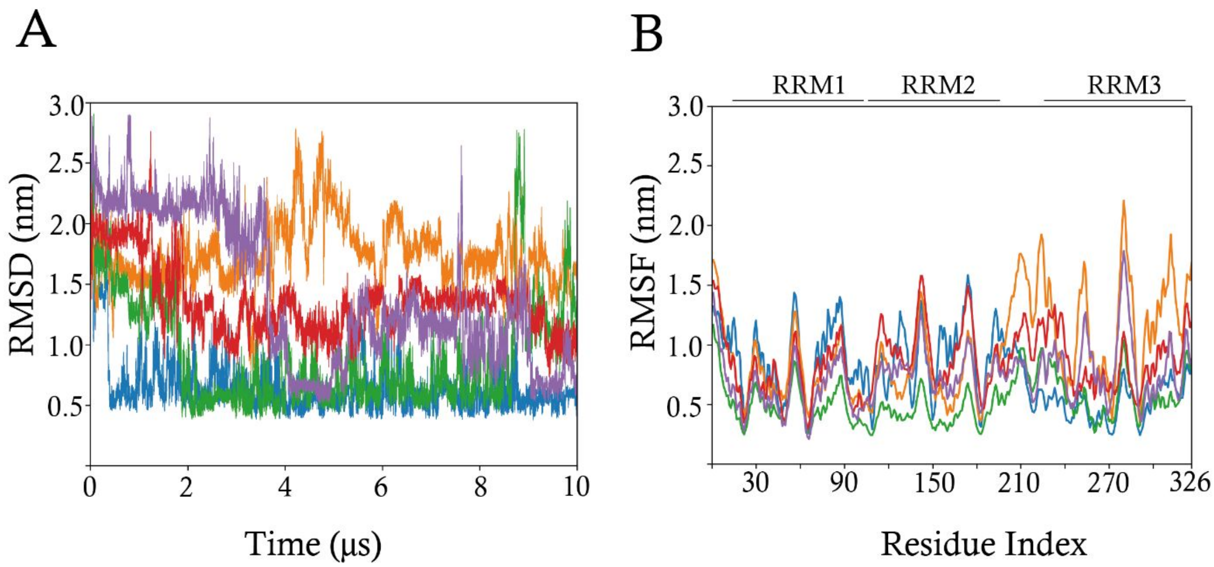

Figure S4. Structural analysis on the CG-HuR MD trajectories. (A) Root Mean Square Deviations of replica 1 (blue), 2 (orange), 3 (green), 4 (red), and 5 (purple). (B) Root Mean Square Fluctuations. Colors are assigned according to the scheme reported in Figure S2. The RRM1, RRM2, and RRM3 domains are highlighted in the plots, as indicated by the labels.

S5. Interdomain analyses between HuR RRM domains over time in CG-MD trajectories.

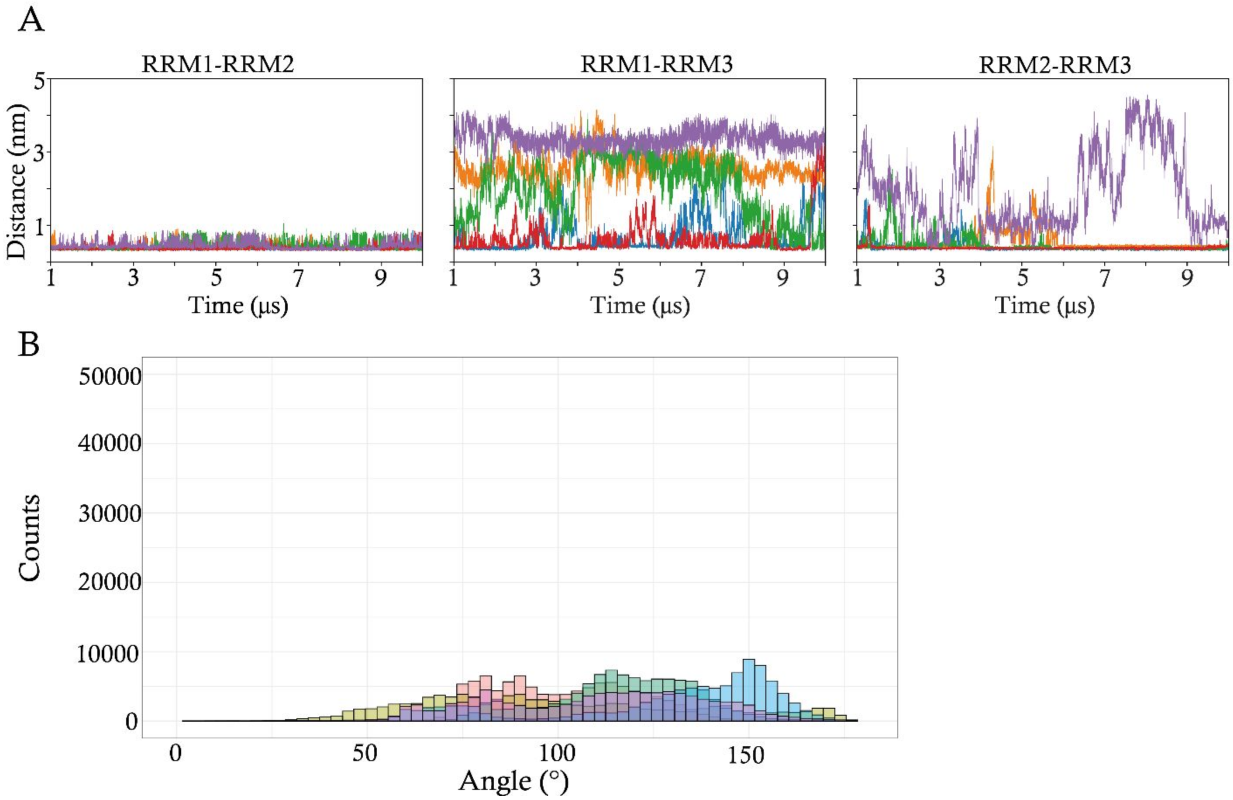

Figure S5. Center of Mass distances between interdomains RRM1-RRM2 (left), RRM1-RRM3 (center), and RRM2-RRM3 (right) for the five replicas. The distances were calculated on all atoms of domains, excluding the hydrogens. Distribution of the angle, expressed in degrees, defined by the centers of mass of the RRM1, RRM2, and RRM3 domains of the CG system (C). Colors follow the scheme reported in Figure S2.

S6. Intramolecular hydrogen bonds network between of the HuR and HuR-mRNA systems.

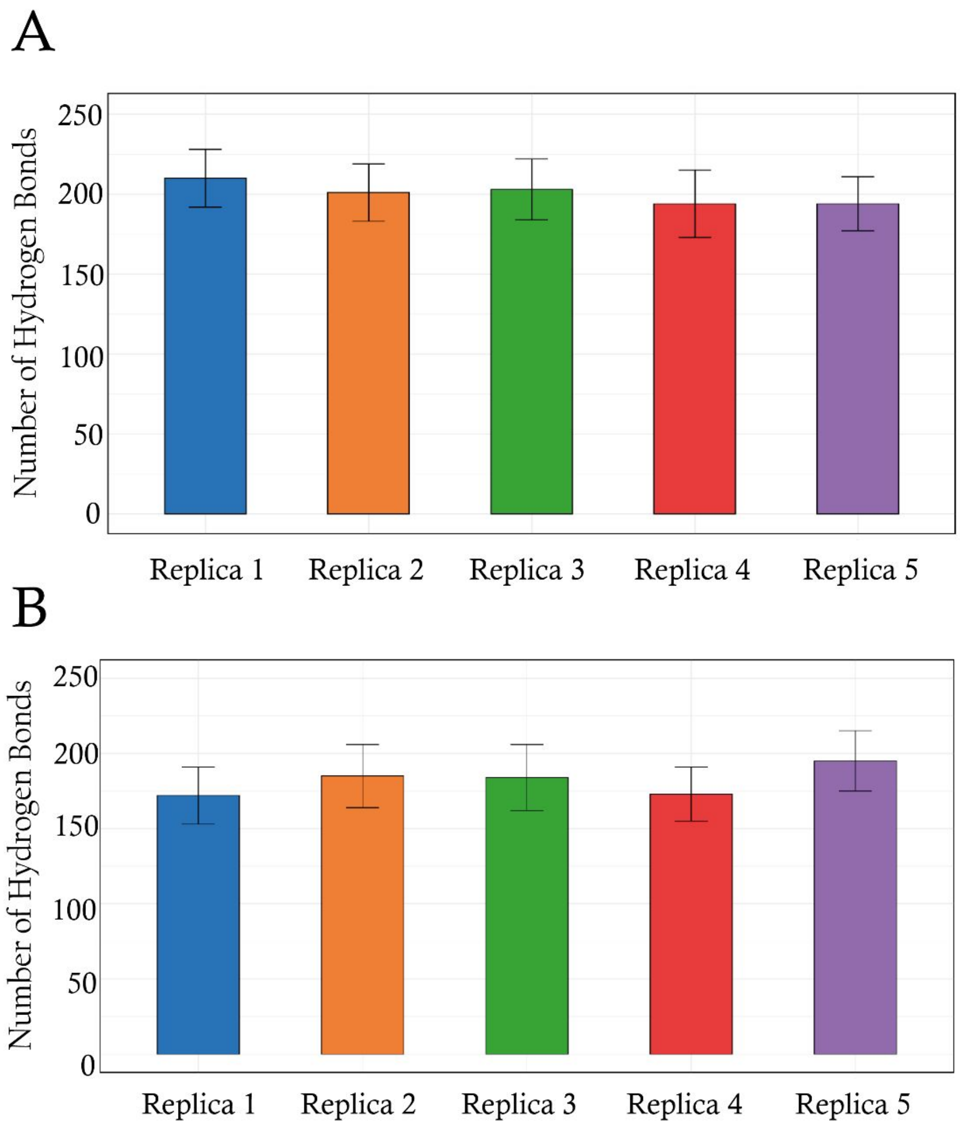

Figure S6. Number of intramolecular hydrogen bonds, expressed as average ± standard deviation, of the (A) unbound HuR and (B) HuR-mRNA systems.

S7. Hydrogen bonds network between the RRM2-RRM3 linker and RRM3 domain region and the RNA of HuR-mRNA systems.

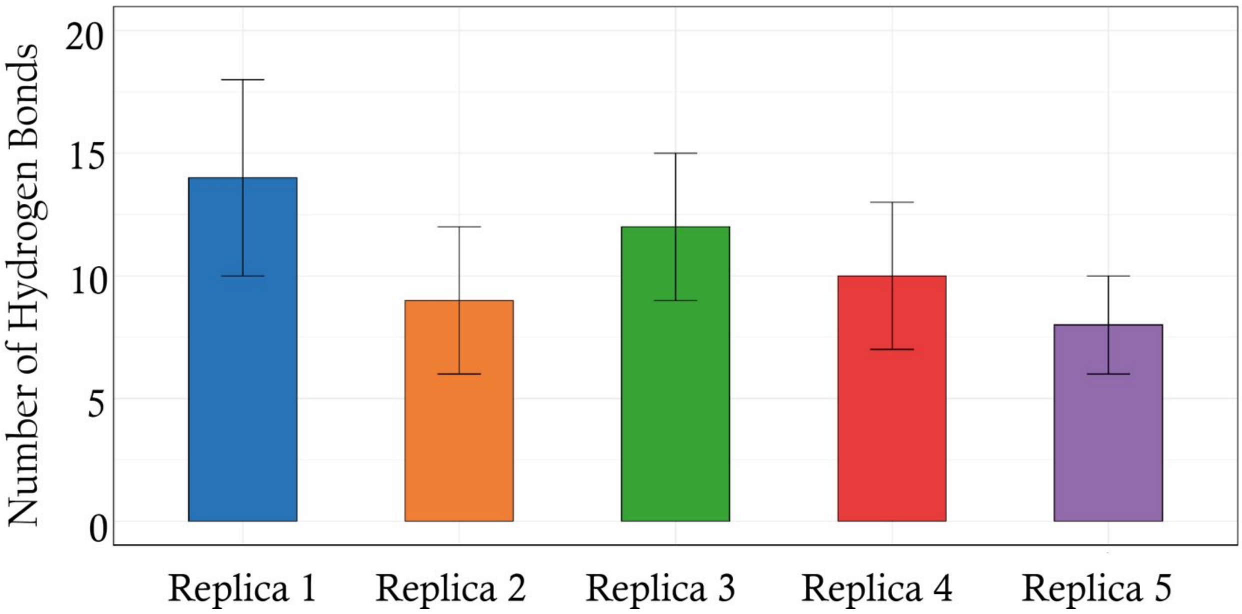

Figure S7. Number of hydrogen bonds, expressed as average ± standard deviation, between the RRM2-RRM3 linker and RRM3 domain region and the RNA of HuR-mRNA systems.

S8. Heatmap of the linker-RRM3 domain region and RNA hydrogen bond pairs.

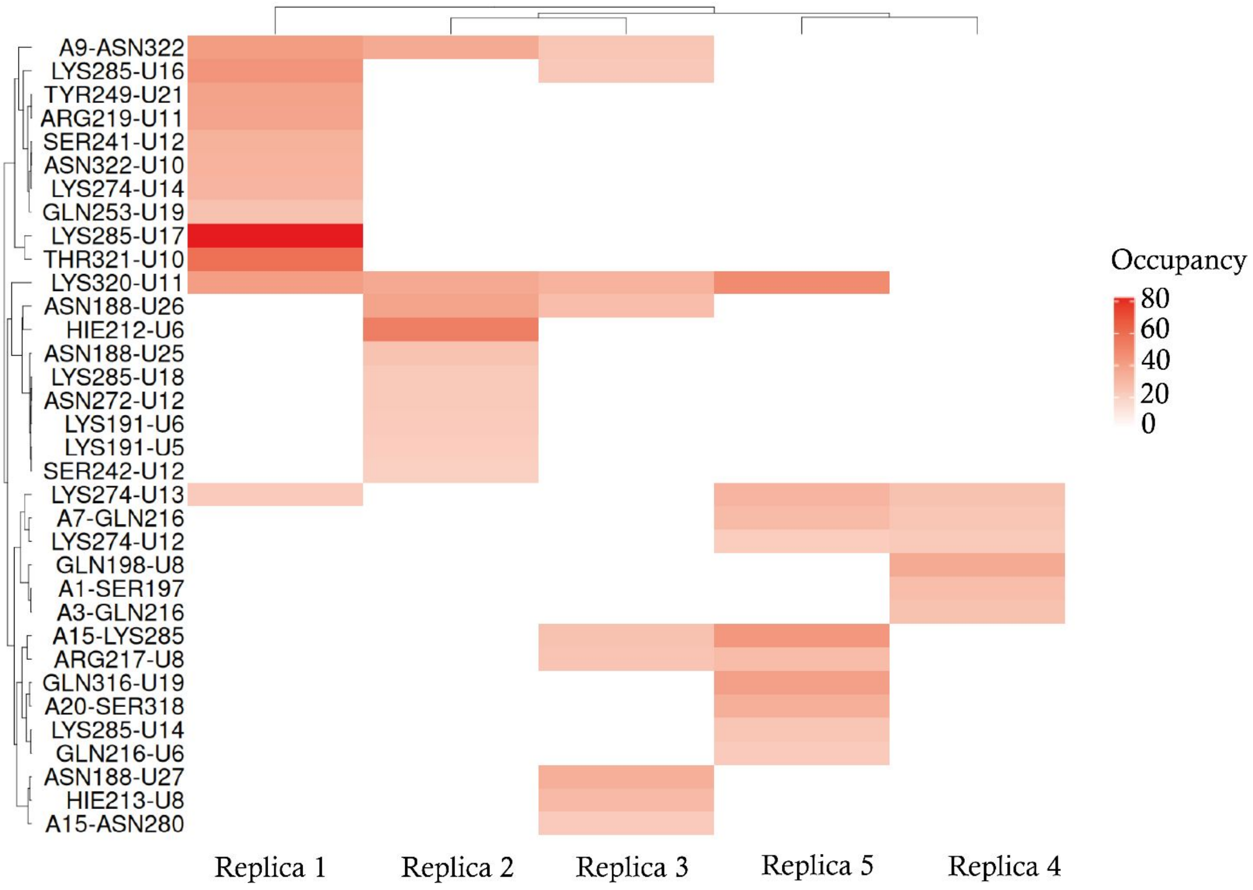

Figure S8. Heatmap of the linker-RRM3 domain region and RNA hydrogen bond pairs, filtered using a cutoff of 20% of occupancy. For each pair, the first residue is the donor, while the second is the acceptor. The colour legend represents the percentage of time occupancy each hydrogen bond is maintained.

S9. Residues of the linker-RRM3 domain region contacting the RNA bases.

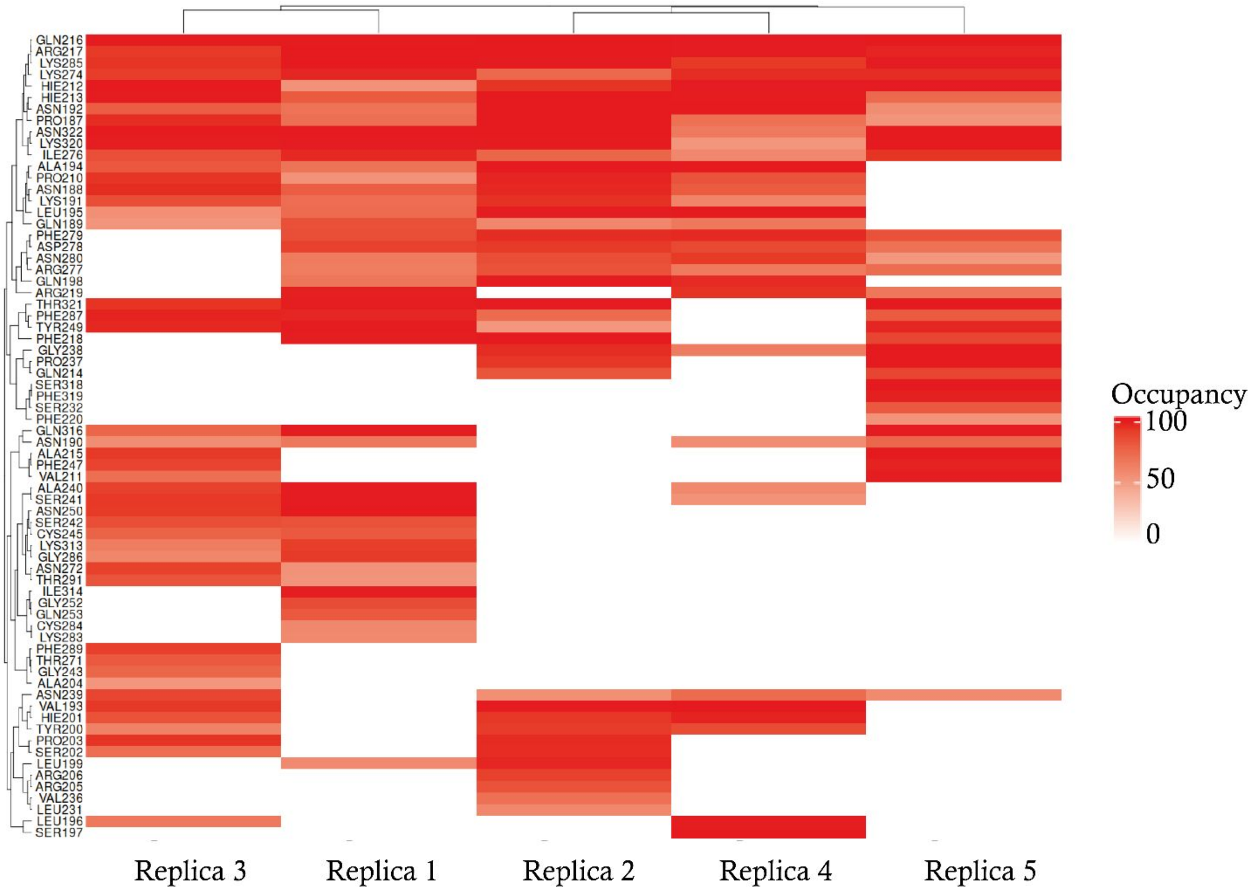

Figure S9 Residues of the linker-RRM3 domain region contacting the RNA bases, filtered with a cutoff of 50% of occupancy. The colour legend represents the percentage of time occupancy each contact is maintained.

S10. MM/PBSA analyses of the MD trajectories of HuR-mRNA systems.

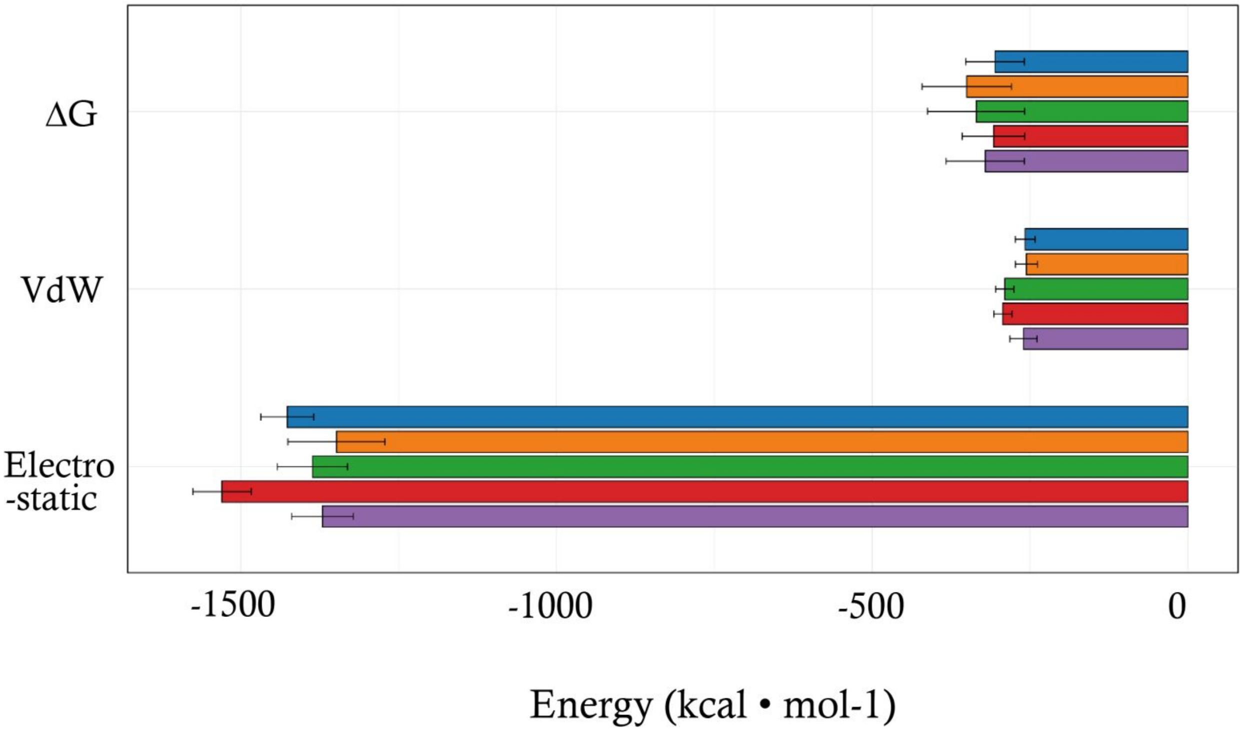

Figure S10. Results of the non-linear MM/PBSA analyses of the MD trajectories of HuR-mRNA systems. The ΔG, Van der Waals component (VdW), and electrostatic component are reported for each system, all expressed in kcal·mol^{-1}. Colors follow the scheme reported in Figure S2.

S11. Protein sequence alignment of Hu family proteins.

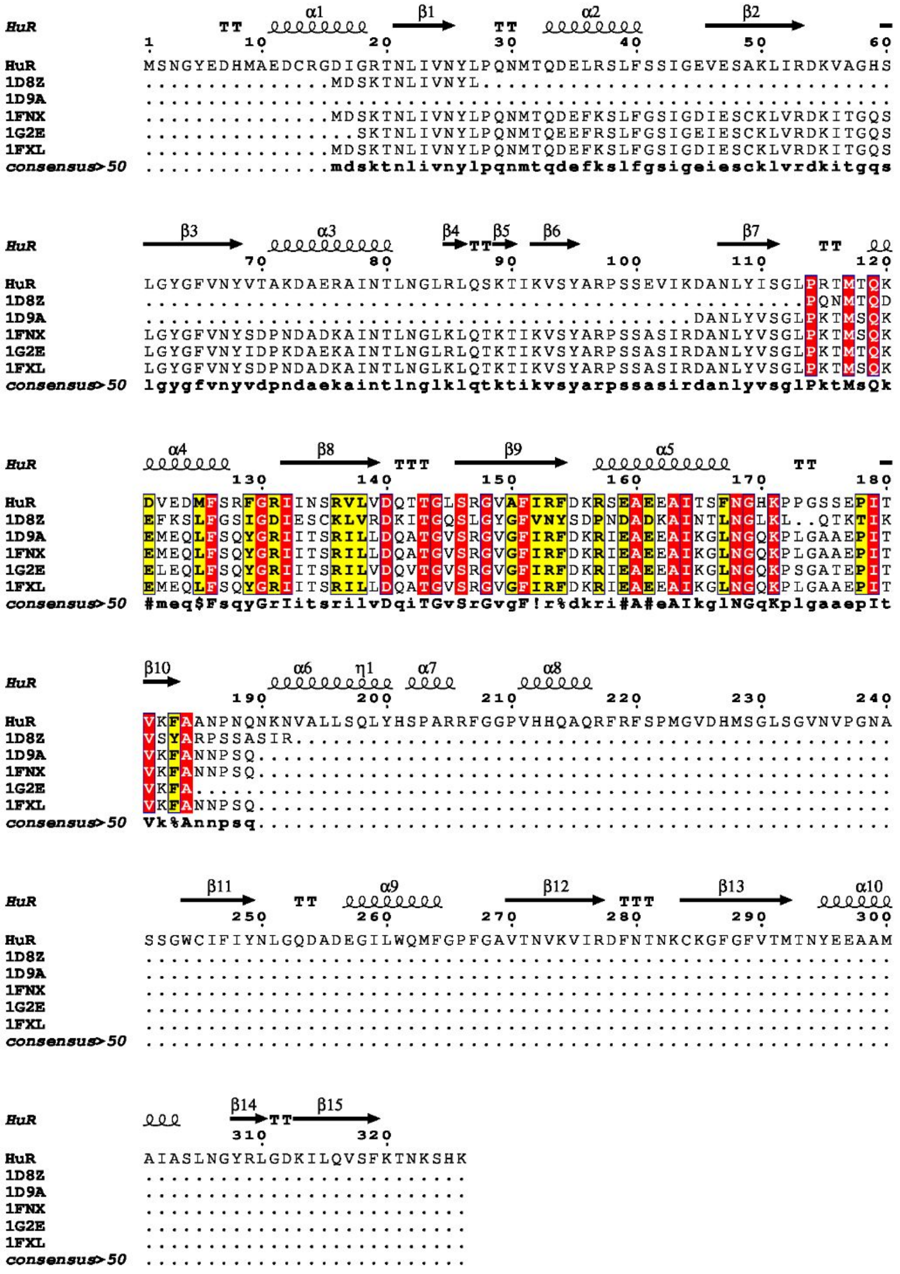

Figure S11. Protein sequence alignment of Hu family proteins. The secondary structure prediction is reported at the upper level. The HuR input sequence was retrieved from the HuR-mRNA complex, after the removal of the mRNA fragment. The PDB IDs 1D8Z, 1D9A and 1FNX correspond to the HuC protein, while the PDB IDs 1G2E and 1FXL correspond to the HuD protein. The colour coding is as follows: red box with white characters indicates strict

sequence identity; black bold characters represent similarity within a group where ISc > ThIn; blue frame with a yellow background denotes similarity across groups where TSc > ThIn; fluorescent green boxes highlight differences between conserved groups where (ISc - XSc) / 2 > ThDiff. The position 26 is specifically marked with a black square. The alignment also includes the predicted secondary structure.

S12. Secondary structure and solvent accessibility predictions for the HuR protein sequence.

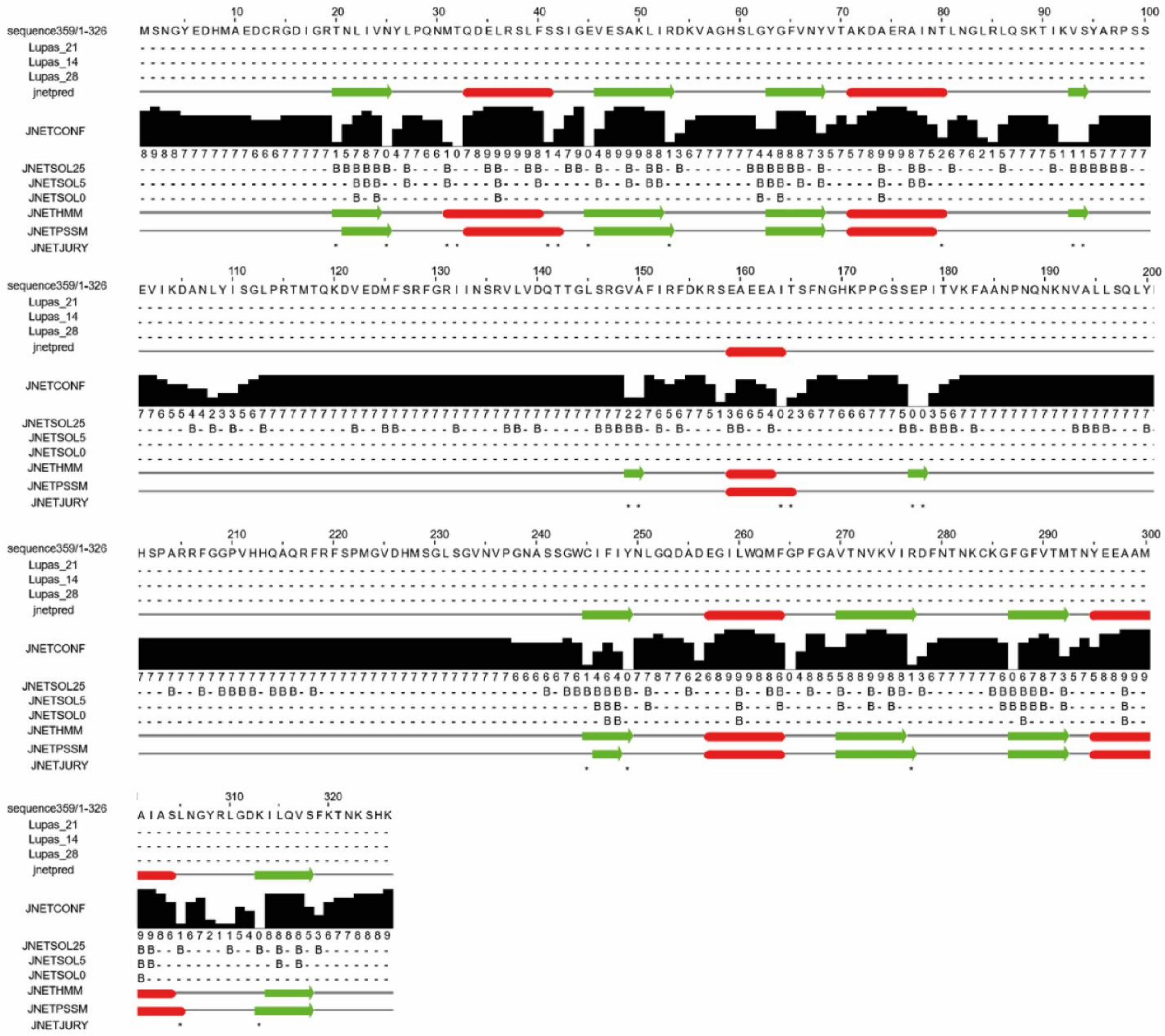

Figure S12. Secondary structure and solvent accessibility predictions for the HuR protein sequence (residues 1-326), obtained from the JPRED web server. For each panel: Lupas_21, Lupas_14, and Lupas_28 refer to the predicted coiled-coil regions, using different window sizes (21, 14, and 28 residues); JNETCONF represents the confidence score for the JNet secondary structure predictions; JNETSOL25, JNETSOL5, and JNETSOL0 represent the solvent accessibility predictions at different thresholds (25% solvent exposure, 5% solvent exposure, and fully buried residues, respectively); JNETHMM provides the secondary structure prediction

using Hidden Markov Models (HMM), which infer structural elements based on statistical patterns in sequence alignments; JNETPSSM refers to the prediction using a Position-Specific Scoring Matrix (PSSM), leveraging evolutionary information to refine secondary structure predictions; JNETJURY represents the consensus prediction that integrates results from different methods, providing a more robust structural assignment.

In the figure, red bars represent α-helices, green arrows indicate β-strands, and black histograms denote confidence scores for secondary structure predictions. Dots and dashes indicate residue-specific solvent accessibility predictions

S13. Structural analysis of the Y26A mutant atomistic molecular dynamics simulations.

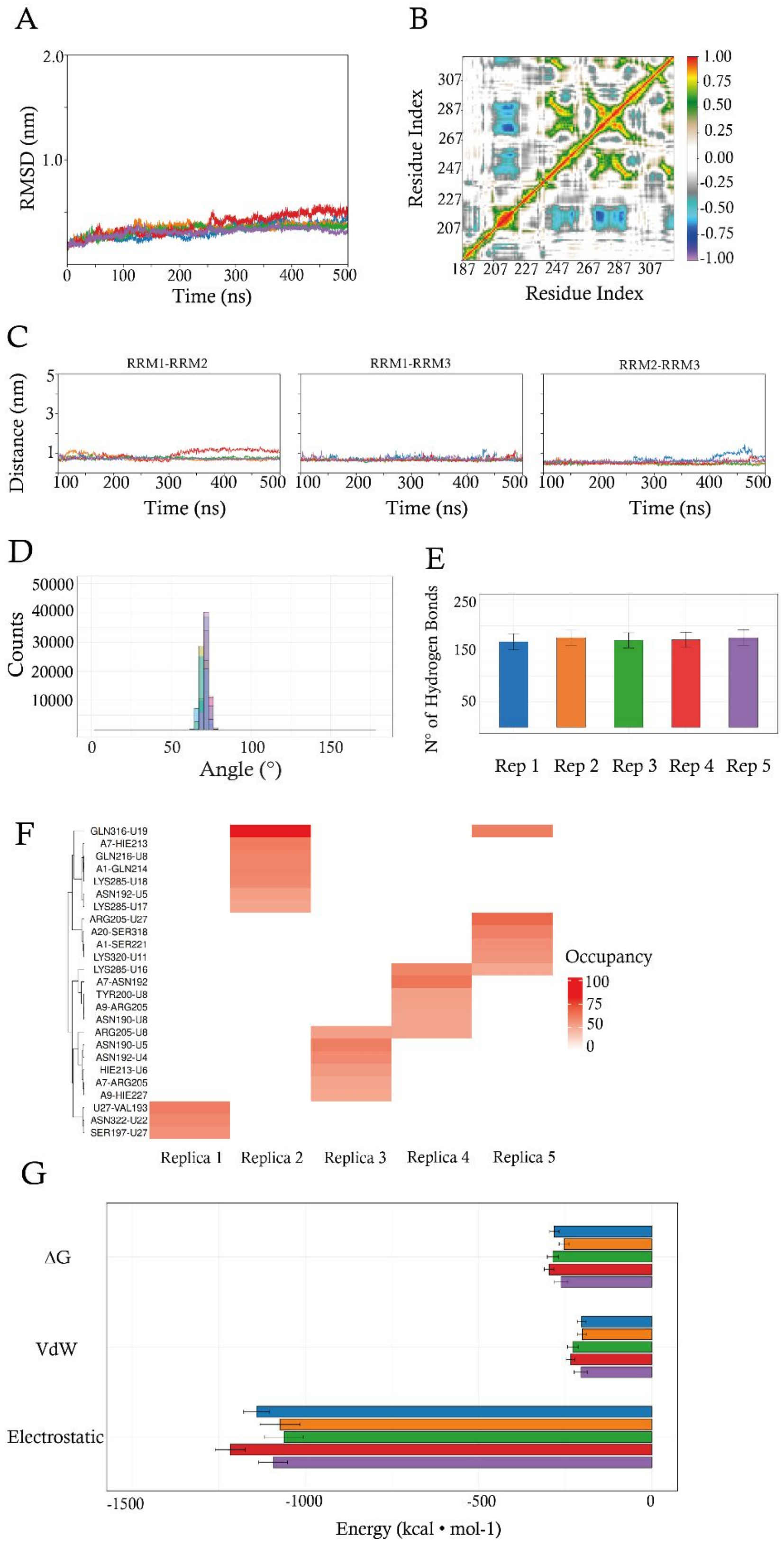

Figure S13. (A) Root Mean Square Deviations (RMSD) of Y26A mutant system for the five replicas. Colors follow the scheme reported in Figure S2. (B) Dynamic Cross Correlation Matrices (DCCM) calculated on the 126 Cα atoms of the RRM2-RRM3 linker and RRM3 region (residues 187-322) of Y26A systems. Color coding is reported in the figure legend. Positive values between two residues indicate a correlated motion, meaning that the residues are moving in the same direction, while negative values indicate an anti-correlated motion, meaning that the residues are moving into different directions. (C) Center of Mass distances between RRM1-RRM2 (left), RRM1-RRM3 (center), and RRM2-RRM3 (right) domains of Y26A system. The distances were calculated on all atoms of domains, excluding the hydrogens. Colors follow the scheme reported in panel A. (D) Distribution of the angle, expressed in degrees, defined by the centers of mass of the RRM1, RRM2, and RRM3 domains of the CG system (C). Colors follow the scheme reported in panel A. (E) Number of intramolecular hydrogen bonds, expressed as average ± standard deviation, of the Y26A systems. (F) Heatmap of the linker-RRM3 domain region and RNA hydrogen bond pairs, filtered using a cutoff of 20% of occupancy. For each pair, the first residue is the donor, while the second is the acceptor. The colour legend represents the percentage of occupancy of each hydrogen bonds. (G) Results of the non-linear MM/PBSA analyses of the MD trajectories of HuR-mRNA systems. The Van der Waals component (VdW), electrostatic component and the ΔG are reported for each system, all expressed in kcal·mol^{-1}.
